# First Total Synthesis of 5′-*O*-α-d-Glucopyranosyl Tubercidin

**DOI:** 10.3390/md18080398

**Published:** 2020-07-29

**Authors:** Wenliang Ouyang, Haiyang Huang, Ruchun Yang, Haixin Ding, Qiang Xiao

**Affiliations:** Jiangxi Key Laboratory of Organic Chemistry, Institute of Organic Chemistry, Jiangxi Science and Technology Normal University, Nanchang 330013, China; ouyangwl93@yeah.net (W.O.); ouyangruchun@163.com (R.Y.); dinghaixin_2010@163.com (H.D.)

**Keywords:** total synthesis, natural product, 7-deazapurine nucleoside, disaccharide nucleoside, tubercidin

## Abstract

The first total synthesis of 5′-*O*-α-d-glucopyranosyl tubercidin was successfully developed. It is a structurally unique disaccharide 7-deazapurine nucleoside exhibiting fungicidal activity, and was isolated from blue-green algae. The total synthesis was accomplished in eight steps with 27% overall yield from commercially available 1-*O*-acetyl-2,3,5-tri-*O*-benzoyl-β-d-ribose. The key step involves stereoselective α-*O*-glycosylation of the corresponding 7-bromo-6-chloro-2′,3′-*O*-isopropylidene-β-d-tubercidin with 2,3,4,6-tetra-*O*-benzyl-glucopyranosyl trichloroacetimidate. All spectra are in accordance with the reported data for natural 5′-*O*-α-d-glucopyranosyl tubercidin. Meanwhile, 5′-*O*-β-d-glucopyranosyl tubercidin was also prepared using the same strategy.

## 1. Introduction

**Figure 1:**
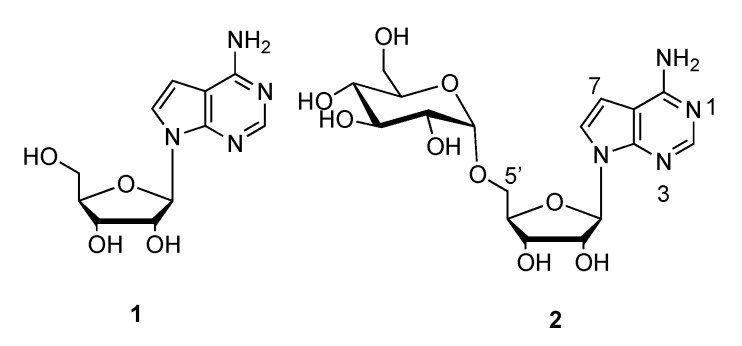
Chemical structures of tubercidin (**1**) and 5′-*O*-α-d-glucopyranosyl tubercidin (**2**).

As the first isolated naturally occurring 7-deazapurine nucleoside, tubercidin (Figure 1, **1**) is a biogenic analogue of adenosine, in which the *N*-7 atom is replaced by CH (purine numbering is used throughout this work) [1,2,3]. It showed significant cytostatic activity in various cancer cell lines. Although the application of tubercidin in clinical trials has been attempted, its use was eventually halted because of significant toxicity [4,5,6,7]. Since then, 7-deazapurine has been recognized as a privileged scaffold in developing new antitumor and antiviral nucleosides [8]. Consequently, many 7-deazapurine nucleosides have been synthesized and their biological activities have been screened in the past 50 years [9,10,11]. Among them, several promising lead compounds have been discovered [12,13,14].

5′-*O*-α-d-Glucopyranosyl tubercidin 1 is a disaccharide nucleoside isolated from blue-green algae in 1988 (Figure 1, **2**) [15]. It is characterized by a unique 5′-*O*-α-d-glucopyranosyl moiety attached to tubercidin. Preliminary biological evaluation indicated that it exhibited moderate cytotoxicity and fungicidal activity. Although many disaccharide nucleosides have been identified as naturally occurring products [16,17], disaccharide 7-deazapurine nucleosides are rarely reported.

Our group has a long-standing interest in total synthesis and biological activity evaluation of naturally occurring 7-deazapurine nucleosides and their biological activities [18,19,20,21,22]. Because of the unique disaccharide structure and a great need for structure-based biological study, structural confirmation of 5′-*O*-α-d-glucopyranosyl tubercidin **2** and synthesis of its analogues in a concise and modular fashion are preferred. Herein, we report the first total synthesis of 5′-*O*-α-d-glucopyranosyl tubercidin **1**.

**Scheme 1:**
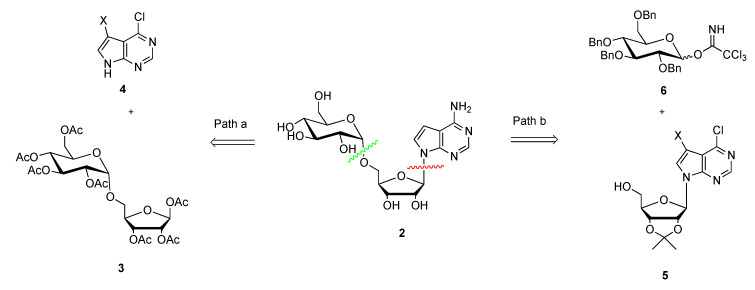
Retrosynthetic analysis of 5′-*O*-α-d-glucopyranosyl tubercidin **2**.

Our retrosynthetic analysis is depicted in Scheme 1. From a synthetic point of view, there are two possible approaches. The first approach is Vorbrüggen glycosylation of disaccharide **3** with nucleobase **4** directly (Scheme 1, Path a). Because of the labile α-*O*-glycosylic bond of disaccharide **3**, the isomerization might occur during Vorbrüggen glycosylation. The second approach is postglycosylation of properly protected nucleoside **5** with glycosyl donor **6** (Scheme 1, Path b). The postglycosylation approach has been widely used for synthesizing disaccharide nucleosides [23,24,25]. Considering that a modular strategy is needed, we employed the postglycosylation approach herein.

## 2. Results

**Scheme 2:**
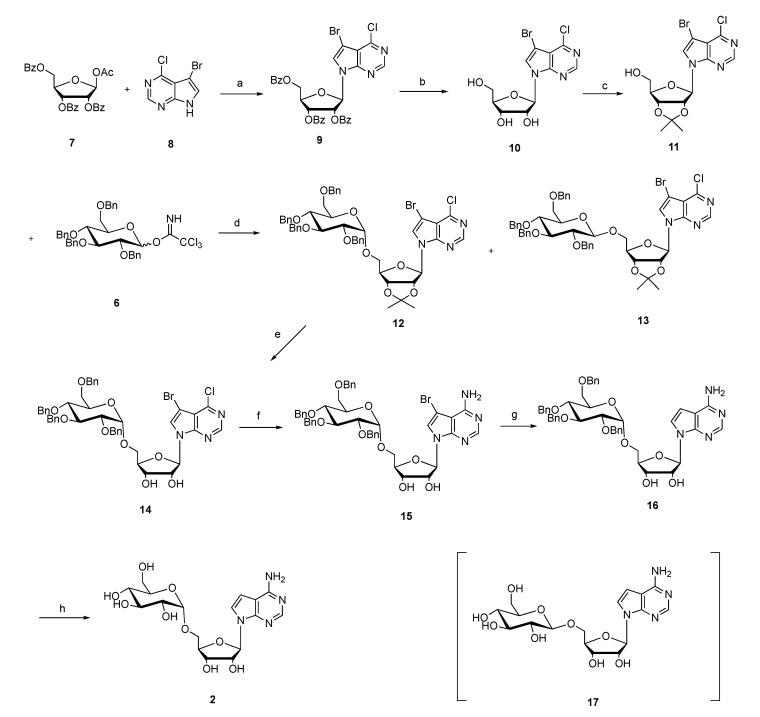
Total synthesis of 5′-*O*-α-d-glucopyranosyl tubercidin **2**. Reagents and conditions: (**a**) bis(trimethylsilyl)acetamide (BSA), TMSOTf, CH_3_CN, 80 °C, 1 h, 75%; (**b**) NH_3_, MeOH, 0 °C, 12 h, 94%; (**c**) 2,2-dimethoxy propane, p-toluenesulfonic acid, acetone, rt., 4 h, 98%; (**d**) TMSOTf, CH_2_Cl_2_, −20 °C, 4 h, 79% (α:β, 4:1); (**e**) 80% aqueous acetic acid, 50 °C, 12 h, 85%; (**f**) NH_3_, MeOH, 130 °C, 12 h, 95%; (**g**) H_2_, 10% Pd/C, Et_3_N, THF/MeOH, rt. 5 h, 83.5%; (**h**) H_2_, 20% Pd(OH)_2_/C, THF/MeOH, 40 °C, 12 h, 80%.

First, 7-deazpurine nucleoside **9** was synthesized based on a previous report by Seela and coworkers (Scheme 2) [26,27,28,29]. After silylation of 6-chloro-7-bromo-7-deazapurine **8** with *N*,*O*-bis(trimethylsilyl)acetamide (BSA) in freshly distilled acetonitrile (CH_3_CN), 1-*O*-acetyl-2,3,5-tri-*O*-benzoyl-β-d-ribose **7** (2 eq.) was added followed by TMSOTf (3 eq.) in one pot. Then, the resulting reaction mixture was heated at 80 °C for 8 h to afford 7-deazpurine nucleoside **9** with a 76% yield. The 7-bromo substitute is critical for the above Vorbrüggen glycosylation, otherwise the reaction cannot proceed. The reason may be that the 7-bromo substituent can reduce the reactivity of *N*-9, which results in the deazapurine being more purine-like [20,26,27,28,29].

Then, all benzoyl groups of nucleoside **9** were removed in saturated ammonia in methanol at 0 °C to give nucleoside **10** in a 94% yield. It should be noted that the C-6 chloride was retained to avoid extra manipulation of protecting groups in the following steps. Next, *p*-toluenesulfonic acid-catalyzed reaction of nucleoside **10** with 2, 2-dimethoxy propane in acetone afforded isopropylidene-protected nucleoside **11** with a 98% yield, which left the 5′-OH free for further glycosylation.

With nucleoside **11** in hand, we focused on carrying out the key glycosylation reaction. In general, 1,2-*trans* β-*O*-glucoside can be prepared by the neighboring group participation of the 2-*O*-acyl glucose donor, which is very reliable and highly stereoselective [29]. In contrast, the construction of our desired 1,2-*ci*s α-*O*-glucoside is much more challenging. It requires glycosyl donors having nonassisting functionality at C-2 position, and even so, the reaction normally produces a mixture of α and β isomers [30,31,32]. In this work, we chose 2,3,4,6-tetra-*O*-benzyl-glucopyranosyl trichloroacetimidate **6** as the glucosyl donor, which was synthesized from 2,3,4,6-tetra-*O*-benzyl glucose with trichloroacetonitrile in dichloromethane (DCM) using anhydrous potassium carbonate as catalysis (98% yield, α:β ≈ 1:4) [33,34]. After optimization (Supplementary Materials), TMSOTf catalyzed glycosylation of nucleoside **11** with glycosyl donor **6** at −30 °C gave a mixture of α isomer **12** and β isomer **13** (4:1) in 79% overall yield. Fortunately, they can be separated by careful silica gel chromatography to afford the desired nucleoside **12** with α glycosylic configuration. These two isomers can be clearly identified by the *J*_1″-2″_ coupling constant (α isomer 3.5 Hz, β isomer 8.0 Hz) and ^13^C chemical shift of C1^″^ (α isomer 96.4 ppm, β isomer 102.9 ppm).

Next, the isopropylidene-protecting group was removed in 80% aqueous acetic acid to give nucleoside **14** with an 85% yield. Then, the substitution of C-6 chloride with freshly prepared saturated ammonium in methanol at 130 °C afforded nucleoside **15** with a 95% yield. Then, we tried to remove the C-7 bromide atom and all benzyl-protecting groups by hydrogenation with 10% Pd/C at the same time. However, the presence of triethylamine in the reaction due to the need to neutralize HBr, which is generated through bromide reduction, prevented the removal of the benzyl group. Only bromide was reduced, to afford nucleoside **16** in 84% yield. Finally, further hydrogenation with 20% Pd(OH)_2_/C gave 5′-*O*-α-d-glucopyranosyl tubercidin **2** with 80% yield. All spectra were in accordance with the reported data of the authentic naturally occurring product.

Since the corresponding 5′-*O*-β-d-glucopyranosyl tubercidin has not been previously reported, its synthesis provides more evidence for structural elucidation and biological activity. 5′-*O*-β-d-Glucopyranosyl tubercidin **17** was also prepared according to a similar procedure for the synthesis of nucleoside **2**.

## 3. Materials and Methods

All reagents were purchased from commercial sources and used without purification unless specified. Acetonitrile, pyridine and CH_2_Cl_2_ were refluxed with CaH_2_ and distilled prior to use. THF was dried with LiAlH_4_ and distilled prior to use. Thin-layer chromatography was performed using silica gel GF-254 plates (Qingdao Chemical Company, Qingdao) detected by UV (254 nm) or charting with 10% sulfuric acid in ethanol. Column chromatography was performed on silica gel (200–300 mesh, Qingdao Chemical Company, Qingdao). NMR spectra were recorded on a Bruker AV400 spectrometer. Chemical shifts (*δ*) are reported in ppm and coupling constants (*J*) are reported in Hz. Then, ^1^H and ^13^C NMR spectra were calibrated with TMS as an internal standard. The specific rotation was measured on a Rudolph autopol IV polarimeter. ESI-MS was acquired with a Bruker Dalton microTOFQ II spectrometer.

### 3.1. H NMR, ^13^C NMR and HRMS of 2,3,4,6-O-Tetrabenzyl-d-glucopyranose

R_f_ = 0.32 (PE/EA = 3:1); mp 153–155 °C; ^1^H NMR (400 MHz, CDCl_3_): δ 7.36–7.28 (m, 18H, Bn), 7.18–7.12 (m, 2H, Bn), 5.31–5.19 (m, 1H, H-1), 4.98–4.48 (m, 8H, 4CH_2_), 4.09–3.94 (m, 2H, H-4,5), 3.74–3.58 (m, 4H, H-2,3,6), 3.06 (d, *J* = 2.2 Hz, 1H, 1-OH); ^13^C NMR (101 MHz, CDCl_3_): δ 138.7 (C), 138.2 (C), 137.8 (2C), 128.5 (CH), 128.4 (CH), 128.2 (CH), 128.1 (CH), 128.0 (CH), 127.9 (CH), 127.7 (CH), 127.6 (CH), 91.3 (CH-1), 81.7 (CH-5), 80.0 (CH-2), 77.7 (CH-3), 75.7 (CH_2_), 75.0 (CH_2_), 73.5 (CH_2_), 73.3 (CH_2_), 70.3 (CH-4), 68.6 (CH_2_-6); HRMS Calcd. For C_34_H_36_O_6_Na^+^ [M + Na]^+^: 563.2404, found: 563.2410.

### 3.2. Synthesis of 2,3,4,6-Tetra-O-benzyl-glucopyranosyl Trichloroacetimidate *(**6**)*

To the suspension of anhydrous K_2_CO_3_ (10 g, 101 mmol) in dry dichloromethane (100 mL) was added 2,3,4,6-*O*-tetrabenzyl-d-glucopyranose (10 g, 18.5 mmol) and trichloroacetonitrile (11.3 mL, 112 mmol) sequentially. The mixture was stirred at room temperature for 4 h until the reaction was completed (monitored by TLC, PE/EA = 6:1, R_f_ = 0.21). After evaporation of the solvent under reduced pressure, the crude product was directly purified by flash column chromatography on silica gel (Et_3_N/PE/EA = 0.1:3:1) to provide 12.4 g compound **6** as a white solid (98% yield, α:β ≈ 1:4); mp: 77–79 °C; ^1^H NMR (400 MHz, CDCl_3_): δ 8.73 (s, 0.8H, β-NH), 8.60 (s, 0.2H, -NH), 7.32 (m, 18H), 7.20 (m, 2H), 6.55 (d, *J* = 3.2 Hz, 0.2H, α-H-1), 5.84 (d, *J* = 6.1 Hz, 0.8H, β-H-1), 5.01–4.46 (m, 9H), 3.81–3.76 (m, 4H), 3.67 (d, *J* = 4.6 Hz, 1H); ^13^C-NMR (101 MHz, CDCl_3_): δ 161.2 (C), 138.4 (C), 138.1 (C), 138.0 (C), 138.0 (C), 128.5 (CH), 128.4 (CH), 128.4 (CH), 128.4 (CH), 128.1 (CH), 128.0 (CH), 128.0 (CH), 127.9 (CH), 127.8 (CH), 127.7 (CH), 127.6 (CH), 127.6 (CH), 98.4 (CH), 84.6 (CH), 81.0 (CH), 76.8 (CH), 75.9 (CH_2_), 75.6 (CH_2_), 75.0 (CH_2_), 74.9 (CH_2_), 73.4 (CH), 68.2 (CH); HRMS Calcd. For C_36_H_36_Cl_3_NO_6_Na^+^ [M + Na]^+^: 706.1500, found: 706.1509.

### 3.3. Synthesis of 7-Bromo-6-chloro-pyrrole [2,3-d]pyrimidine *(**8**)*

To the stirred suspension of 6-chloro-pyrrole[2,3-d]pyrimidine (12.0 g, 78.2 mmol) in dry dichloromethane (500 mL) was added NBS (16.1 g, 91.1 mmol) in batches. After the addition was finished, the mixture was stirred at room temperature for another 10 h until completion (monitored by TLC, DCM/EA = 5:1, R_f_ = 0.41) and then poured into ice water (500 mL). The resulting brown precipitate was filtered under reduced pressure and the filter cake was washed with water (500 × 3 mL) and dried to afford compound **8** as a brown solid (17.2 g, 95% yield); mp: >300 °C; ^1^H NMR (400 MHz, DMSO): δ 12.95 (s, 1H, NH), 8.59 (s, 1H, H-2), 7.91 (s, 1H, H-8); ^13^C NMR (100 MHz, DMSO): δ 151.5 (C), 151.4 (CH), 150.7 (C), 129.1 (CH), 114.1 (C), 86.3 (C); HRMS Calcd. For C_6_H_4_BrClN_3_^+^ [M + H]^+^: 231.9272, found: 231.9270.

### 3.4. Synthesis of 7-Bromo-6-chloro-9-(2′,3′,5′-O-tribenzoyl-β-d-ribofuranose-yl)-pyrrole[2,3-d]pyrimidine *(**9**)*

To the stirred suspension of 7-bromo-6-chloro-pyrrole[2,3-d]pyrimidine 8 (5.0 g, 21.5 mmol) in dry acetonitrile (40 mL) was added BSA (*N*,*O*-bis(trimethylsiyl)acetamide, 5.4 g, 26.0 mmol) at 0 °C under argon. The resulting mixture was stirred at room temperature for 15 min, and then 1-*O*-ethanoyl-2,3,5-*O*-tribenzoyl-β-d-ribofuranose (16.0 g, 32.5 mmol) and TMSOTf (8.0 g, 46 mmol) were added sequentially. After the addition was completed, the mixture was stirred at 80 °C for 1 h (monitored by TLC, CH_2_Cl_2_: PE, 5:1, R_f_ 0.27). After the mixture was cooled to room temperature, water (200 mL) was added to quench the reaction and the resulting mixture was extracted with ethyl acetate (200 × 3 mL). The organic layers were separated and washed with saturated NaHCO_3_ (200 mL) and saturated brine (200 mL) and dried with anhydrous Na_2_SO_4_. After the solvent was removed under reduced pressure, the crude product was purified by flash column chromatography on silica gel to provide 10.1 g compound **9** (75.5% yield) as a white solid. mp: 142–143 °C; ${\left[\alpha \right]}_{D}^{25}$ = −110 (*c* = 0.30, CHCl_3_); ^1^H-NMR (400 MHz, CDCl_3_): δ (ppm) 8.57 (s, 1H), 8.10 (d, *J* = 8.0 Hz, 2H), 7.99 (d, *J* = 8.0 Hz, 2H), 7.91 (d, *J* = 7.9 Hz, 2H), 7.61–7.32 (m, 10H), 6.67 (d, *J* = 5.2 Hz, 1H), 6.12 (m, 2H), 4.89 (d, *J* = 12.1 Hz, 1H), 4.80 (d, *J* = 3.1 Hz, 1H), 4.67 (dd, *J* = 12.2, 3.1 Hz, 1H); ^13^C NMR (101MHz, CDCl_3_): δ (ppm) 166.1 (C), 165.4 (C), 165.1 (C), 152.7 (C), 151.6 (CH), 150.7 (C), 133.9 (CH), 133.9 (CH), 133.7 (CH), 129.9 (4CH), 129.7 (2CH), 129.2 (C), 128.8 (2CH), 128.6 (2CH; C), 128.6 (2CH), 128.3 (C), 126.5 (CH), 115.9 (C), 90.2 (C), 86.7 (CH), 80.6 (CH), 74.1 (CH), 71.4 (CH), 63.5 (CH); HRMS Calcd. For C_32_H_23_BrClN_3_O_7_Na^+^ [M + Na]^+^: 698.0300, found: 698.0305.

### 3.5. Synthesis of 7-Bromo-6-chloro-9-(β-d-ribofuranose-yl)-pyrrole[2,3-d]pyrimidine *(**10**)*

7-Bromo-6-chloro-9-(2′,3′,5′-*O*-tribenzoyl-β-d-ribofuranose-yl)-pyrrole[2,3-d]pyrimidine **9** (10 g, 14.77 mmol) was dissolved in methanolic ammonia (methanol was saturated with NH_3_ at 0 °C, 300 mL) and the mixture was stirred at 0 °C for 12 h until the reaction was completed (monitored by TLC, CH_2_Cl_2_:CH_3_OH, 50:1, R_f_ 0.45). The mixture was concentrated and the crude product was purified by flash column chromatography on silica gel (CH_2_Cl_2_/CH_3_OH, 70:1) to provide 5.1 g compound **10** (94.0% yield) as a white solid. mp: 179–180 °C; ${\left[\alpha \right]}_{D}^{25}$ = −64 (*c* = 0.1, CH_3_OH); ^1^H NMR (400 MHz, DMSO): δ 8.72 (s, 1H, H-2), 8.27 (s, 1H, H-8), 6.24 (d, *J* = 5.8 Hz, 1H, H-1′), 5.49 (d, *J* = 6.1 Hz, 1H, 2′-OH), 5.25 (d, *J* = 4.9 Hz, 1H, 3′-OH), 5.15 (t, *J* = 5.3 Hz, 1H, 5′-OH), 4.40 (dd, *J* = 11.1, 5.6 Hz, 1H, H-2′), 4.13 (dd, *J* = 8.3, 4.5 Hz, 1H, H-3′), 3.96 (d, *J* = 3.4 Hz, 1H, H-4′), 3.71–3.56 (m, 2H, H-5′); ^13^C NMR (101 MHz, DMSO): δ 151.6 (CH-2), 151.0 (C-6), 151.0 (C-4), 128.75(CH-8), 114.9 (C-5), 87.7 (C-7; CH-1′), 86.0 (CH-4′), 74.9 (CH-2′), 70.8 (CH-3′), 61.7 (CH_2_-5′); HRMS Calcd. For C_11_H_11_BrClN_3_O_4_Na^+^ [M + Na]^+^: 385.9514, found: 385.9511.

### 3.6. Synthesis of 7-Bromo-6-chloro-9-(2′,3′-O-isopropylidene-β-d-ribofuranose-yl)-pyrrole[2,3-d] pyrimidine *(**11**)*

To the stirred suspension of compound **10** (4.0 g, 11 mmol) in dry acetone (200 mL) was added *p*-toluenesulfonic acid (200 mg, 1.2 mmol) and 2,2-dimethoxy propane (6.0 g, 55 mmol). The mixture was stirred at room temperature for 4 h until the reaction was completed (monitored by TLC, CH_2_Cl_2_:CH_3_OH, 50:1, R_f_ 0.57). After that, the mixture was neutralized by triethylamine (70.0 mg, 0.7 mmol) and concentrated under reduced pressure. The crude product was purified by flash column chromatography on silica gel (CH_2_Cl_2_/CH_3_OH, 70:1) to provide 4.4 g compound **11** (98.0% yield) as a white solid. mp: 73–74 °C; ${\left[\alpha \right]}_{D}^{25}$ = −63 (*c* = 0.05, CH_3_OH); ^1^H NMR (400 MHz, DMSO): δ 8.73 (s, 1H, H-2), 8.24 (s, 1H, H-8), 6.36 (d, *J* = 2.1 Hz, 1H, H-1′), 5.32–5.04 (m, 2H, H-2′,5′-OH), 5.03–4.83 (m, 1H, H-3′), 4.21 (s, 1H, H-4′), 3.56 (s, 2H, H-5′), 1.54 (s, 3H, Me), 1.31 (s, 3H, Me); ^13^C NMR (101 MHz, DMSO): δ 151.8 (CH-2), 151.2 (C-4), 150.5 (C-6), 129.2 (CH-8), 115.0 (C-5), 113.6 (C), 90.0 (CH-1′), 87.8 (C-7), 86.9 (CH-4′), 84.4 (CH-2′), 81.4 (CH-3′), 61.9 (CH_2_-5′), 27.5 (Me), 25.6 (Me); HRMS Calcd. For C_14_H_15_BrClN_3_O_4_Na^+^ [M + Na]^+^: 425.9827, found: 425.9811.

### 3.7. Synthesis of 7-Bromo-6-chloro-(2′,3′-O-isopropylidene-5′-O-(2″,3″,4″,6″-O-tetrabenzyl-α-d-glucopyranosyl)tubercidin (**12**) and 7-bromo-6-chloro-(2′,3′-O-isopropylidene-5′-O-(2″,3″,4″,6″-O-tetrabenzyl-β-d-glucopyranosyl)tubercidin *(**13**)*

Compound **6** (6.4 g, 9.3 mmol) and compound **11** (2.5 g, 6.2 mmol) were dissolved in dry dichloromethane (300 mL) and the resulting mixture was stirred at room temperature for 5 min under argon. After that, TMSOTf (16.5 mg, 0.07 mmol) was added to the mixture at −20 °C. The mixture was slowly warmed to room temperature and stirred for another 4 h until the reaction was completed (monitored by TLC, PE: EA, 3:1, R_f_ 0.5). The mixture was concentrated and the crude product was purified by flash column chromatography on silica gel (CH_2_Cl_2_/CH_3_OH, 70:1) to provide 8.0 g of compounds **12** and **13** (79% yield) as a light yellow oil.

**12:**${\left[\alpha \right]}_{D}^{25}$ = −7 (*c* = 0.1, CH_3_OH); ^1^H NMR (400 MHz, DMSO): δ 8.71 (s, 1H), 8.23 (s, 1H), 7.34–7.22 (m, 18H), 7.13–7.04 (m, 2H), 6.41 (d, *J* = 2.6 Hz, 1H), 5.20 (dd, *J* = 6.1, 2.7 Hz, 1H), 4.97 (dd, *J* = 6.1, 3.2 Hz, 1H), 4.90 (m, 2H), 4.72–4.61 (m, 4H), 4.40 (m, 4H), 3.78–3.67 (m, 2H), 3.61 (dd, *J* = 10.9, 4.1 Hz, 1H), 3.56–3.37 (m, 5H), 1.54 (s, 3H), 1.29 (s, 3H); ^13^C NMR (101 MHz, DMSO): δ 151.8 (CH), 151.3 (C), 150.4 (C), 139.3 (C), 138.9 (C), 138.7 (C), 138.6 (CH), 129.2 (CH), 128.7 (CH), 128.6 (CH), 128.2 (CH), 128.0 (CH), 128.0 (CH), 127.9 (CH), 127.8 (CH), 115.1 (C), 114.0 (C), 96.4 (CH), 89.6 (CH), 88.3 (C), 84.5 (CH), 84.3 (CH), 81.5 (CH), 81.4 (CH), 79.8 (CH), 77.7 (CH), 75.0 (CH_2_), 74.4 (CH_2_), 72.8 (CH_2_), 71.8 (CH_2_), 70.4 (CH), 68.8 (CH_2_), 68.0 (CH_2_), 27.5 (Me), 25.8 (Me); HRMS Calcd. For C_48_H_49_BrClN_3_O_9_Na^+^ [M + Na]^+^: 948.2233, found: 948.2236.

**13:**${\left[\alpha \right]}_{D}^{25}$ = −19 (*c* = 0.1, CH_3_OH); ^1^H NMR (400 MHz, DMSO): δ 8.67 (s, 1H), 8.17 (s, 1H), 7.24 (m, 14H), 7.14 (m, 6H), 6.36 (d, *J* = 2.5 Hz, 1H), 5.19 (dd, *J* = 6.1, 2.6 Hz, 1H), 4.92 (dd, *J* = 6.1, 2.8 Hz, 1H), 4.78–4.64 (m, 4H), 4.56–4.30 (m, 6H), 4.04–3.98 (m, 1H), 3.76–3.38 (m, 6H), 3.24 (m, 1H), 1.50 (s, 3H), 1.23 (s, 3H); ^13^C NMR (101 MHz, DMSO): δ 151.7 (CH), 151.2 (C), 150.3 (C), 139.0 (C), 138.9 (C), 138.7 (C), 138.6 (C), 129.3 (CH), 128.7 (CH), 128.6 (CH), 128.4 (CH), 128.2 (CH), 128.0 (CH), 128.0 (CH), 127.9 (CH), 127.9 (CH), 127.8 (CH), 115.1 (C), 113.8 (C), 102.9 (CH), 90.4 (CH), 87.9 (C), 85.0 (CH), 84.4 (CH), 84.1 (CH), 82.1 (CH), 81.4 (CH), 78.1 (CH), 74.9 (CH_2_), 74.4 (CH_2_), 74.4 (CH_2_), 74.0 (CH_2_), 72.7 (CH), 69.7 (CH_2_), 69.2 (CH_2_), 27.4 (Me), 25.6 (Me); MS (ESI): HRMS Calcd. For C_48_H_49_BrClN_3_O_9_Na^+^ [M + Na]^+^: 948.2233, found: 948.2235.

### 3.8. Synthesis of 7-Bromo-6-chloro-5′-O-(2″,3″,4″,6″-O-tetrabenzyl-α-d-glucopyranosyl) Tubercidin *(**14**)*

Compound **12** (1.0 g, 1.08 mmol) was added to 80% acetic acid aqueous solution (50 mL) and the solution was stirred at room temperature for 30 min. After that, the mixture was transferred to preheated oil and stirred at 50 °C for another 12 h until the reaction was completed (monitored by TLC, CH_2_Cl_2_:CH_3_OH,20:1, R*_f_* 0.35). The mixture was concentrated and the crude product was purified by flash column chromatography on silica gel (CH_2_Cl_2_/CH_3_OH, 50:1) to provide 0.9 g compound **14** (85.0% yield) as a white solid. mp: 49–50 °C; ${\left[\alpha \right]}_{D}^{25}$ = −26° (*c* = 0.3, CH_3_OH); ^1^H NMR (400 MHz, DMSO): δ 8.71 (s, 1H, H-2), 8.37 (s, 1H, H-8), 7.42–7.21 (m, 18H, H-Ph), 7.16–7.09 (m, 2H, H-Ph), 6.35 (d, *J* = 6.4 Hz, 1H, H-1′), 5.60 (d, 1H, 2′-OH), 5.37 (s, 1H, 3′-OH), 5.01 (d, *J* = 11.1 Hz, 1H, H-1″), 4.84 (m, 2H, H-CH_2_), 4.77–4.65 (m, 3H, H-CH_2_; H-2′), 4.45 (m, 4H, H-CH_2_), 4.19 (d, *J* = 2.3 Hz, 1H, H-3′), 4.12 (s, 1H, H-5′), 3.82–3.62 (m, 3H, H-5′; H-4′), 3.58–3.56 (m, 3H, H-3″; H-6″), 3.52–3.40 (m, 2H, H-2″; H-4″); ^13^C NMR (101 MHz, DMSO): δ 151.7 (CH-2), 151.3 (C-6), 151.1 (C-4), 139.3 (C), 138.8 (C), 138.6 (2C), 129.6 (CH), 128.7 (CH), 128.6 (CH), 128.5 (CH), 128.4 (CH), 128.2 (CH), 128.1 (CH), 127.9 (CH), 127.89 (CH), 114.9 (C-5), 96.4 (CH-1″), 88.4 (C-7), 87.3 (CH-1′), 83.8 (CH-5″), 82.0 (CH-4′), 79.5 (CH-4″), 77.8 (CH-3″), 75.3 (CH_2_), 75.1 (CH-2′), 74.6 (CH_2_), 72.8 (CH_2_), 71.9 (CH_2_), 71.6 (CH-3′), 70.6 (CH-2″), 69.0 (CH_2_-6″), 67.9 (CH_2_-5′); HRMS Calcd. For C_45_H_46_BrClN_3_O_9_^+^ [M + H]^+^: 886.2100, found: 886.2098.

### 3.9. Synthesis of 7-Bromo-6-chloro-5′-O-(2″,3″,4″,6″-O-tetrabenzyl-β-d-glucopyranosyl) Tubercidin (14′)

Compound **13** was converted into **14′** (79.0% yield) as described for the synthesis of **14**: R_f_ 0.34 (CH_2_Cl_2_/CH_3_OH, 20:1); mp: 49–50 ^°^C; ${\left[\alpha \right]}_{D}^{25}$ = −12 (*c* = 0.3, CH_3_OH); ^1^H NMR (400 MHz, DMSO): δ 8.67 (s, 1H), 8.22 (s, 1H), 7.24 (s, 14H), 7.17–7.05 (m, 6H), 6.23 (d, *J* = 5.4 Hz, 1H), 5.54 (d, *J* = 6.1 Hz, 1H), 5.34 (s, 1H), 4.78 (d, *J* = 11.1 Hz, 2H), 4.69 (d, *J* = 8.8 Hz, 2H), 4.60–4.37 (m, 7H), 4.12 (m, 3H), 3.86–3.72 (m, 1H), 3.72–3.50 (m, 4H), 3.45 (m, 1H), 3.29–3.23 (m, 1H); ^13^C NMR (101 MHz, DMSO) δ 151.6 (CH-2), 151.0 (C-6), 150.9 (C-4), 139.1 (C), 138.8 (C), 138.7 (C), 138.6 (C), 128.8 (CH), 128.7 (CH), 128.6 (CH), 128.6 (CH), 128.5 (CH), 128.4 (CH), 128.2 (CH), 128.1 (CH), 128.0 (CH), 127.9 (CH), 127.9 (CH), 127.8 (CH), 127.8 (CH), 115.0 (C-5), 103.0 (CH-1″), 88.2 (C-7), 87.9 (CH-1′), 84.1 (CH-4′), 83.7 (CH-5″), 82.4 (CH-4″), 78.1 (CH-3″), 74.9 (CH-2′), 74.7 (CH-3′), 74.5 (CH_2_), 74.4 (CH_2_), 74.1 (CH_2_), 72.7 (CH_2_), 71.0 (CH-2″), 70.0 (CH_2_-5′), 69.2 (CH_2_-6″); HRMS Calcd. For C_45_H_46_BrClN_3_O_9_^+^ [M + H]^+^: 886.2100, found: 886.2099.

### 3.10. Synthesis of 7-Bromo-5′-O-(2″,3″,4″,6″-O-tetrabenzyl-α-d-glucopyranosyl)tubercidin *(**15**)*

Compound **14** (100 mg, 0.11 mmol) was dissolved in methanolic ammonia (methanol was saturated with NH_3_ at 0 °C, 50 mL) and the mixture was stirred at 130 °C for 12 h (monitored by TLC, CH_2_Cl_2_:CH_3_OH, 25:1, R_f_ 0.36). After cooling to room temperature, the mixture was concentrated and the crude product was purified by flash column chromatography on silica gel (CH_2_Cl_2_/CH_3_OH, 30:1) to provide 92.9 mg compound **15** (94.9% yield) as a white solid. mp: 68–69 °C, ${\left[\alpha \right]}_{D}^{25}$ = −33 (*c* = 0.1, CH_3_OH); ^1^H NMR (400 MHz, DMSO): δ 8.10 (s, 1H, H-2), 7.87 (s, 1H, H-8), 7.40–7.18 (m, 18H, H-Bn), 7.18–7.07 (m, 2H, H-Bn), 6.76 (br s, 2H, H-NH_2_), 6.19 (d, *J* = 6.6 Hz, 1H, H-1′), 5.46 (d, *J* = 6.3 Hz, 1H, 2′-OH), 5.26 (d, *J* = 4.1 Hz, 1H, 3′-OH), 4.98 (d, *J* = 11.1 Hz, 1H, H-1″), 4.81–4.66 (m, 5H, H-2′; 2CH_2_), 4.49–4.32 (m, 4H, 2CH_2_), 4.10–4.09 (m, 1H, H-3′), 4.06–4.04 (m, 1H, H-5″), 3.78–3.50 (m, 6H, H-4′,5′,3″,6″), 3.48–3.39 (m, 2H, H-2″,4″); ^13^C NMR (101 MHz, DMSO): δ 157.4 (C-4), 153.0 (C-6), 150.6 (CH-2), 139.3 (C), 138.8 (C), 138.7 (2C), 128.7 (CH), 128.7 (CH), 128.4 (CH), 128.2 (CH), 128.1 (CH), 128.0 (CH), 127.9 (CH), 121.7 (C-5), 96.3 (CH-1″), 87.9 (C-7), 86.5 (CH-1′), 83.3 (CH-5″), 82.0 (CH-4′), 79.5 (CH-4″), 77.8 (CH-3″), 75.3 (CH_2_), 74.9 (CH-2′), 74.6 (CH_2_), 72.8 (CH_2_), 71.8 (CH_2_), 71.7 (CH-3′), 70.6 (CH-2″), 69.0 (CH_2_-6′), 68.1 (CH_2_-5′); HRMS Calcd. For C_45_H_48_BrN_4_O_9_^+^ [M + H]^+^: 867.2599, found: 867.2598.

### 3.11. Synthesis of 7-Bromo-5′-O-(2″,3″,4″,6″-O-tetrabenzyl-β-d-glucopyranosyl) Tubercidin *(**15′**)*

Compound **14′** was converted into **15′** (79.0% yield) as described for the synthesis of **15**; R_f_ 0.40 (CH_2_Cl_2_/CH_3_OH, 20:1); mp: 145–147 °C; ${\left[\alpha \right]}_{D}^{25}$ = −15 (*c* = 0.1, CH_3_OH); ^1^H NMR (400 MHz, DMSO) δ 8.11 (s, 1H, H-2), 7.68 (s, 1H, H-8), 7.40–7.19 (m, 14H, H-Bn), 7.19–7.10 (m, 6H, H-Bn), 6.77 (br s, 2H, NH_2_), 6.13 (d, *J* = 5.4 Hz, 1H, H-1′), 5.44 (d, *J* = 6.1 Hz, 1H, 2′-OH), 5.26 (d, *J* = 5.0 Hz, 1H, 3′-OH), 4.81 (m, 2H, H-2′,1″), 4.71 (d, *J* = 11.0 Hz, 2H, CH_2_), 4.60–4.08 (m, 6H, 3CH_2_), 4.15–4.04 (m, 3H, H-3′,5′,5″), 3.78–3.42 (m, 6H, H-4′,5′,2″,3″,6″), 3.29 (s, 1H, H-4″); ^13^C NMR (101 MHz, DMSO) δ 157.4 (C-6), 153.0 (CH-2), 150.2 (C-4), 139.1 (CH), 138.8 (CH), 138.7 (CH), 138.6 (CH), 128.7 (CH), 128.7 (CH), 128.6 (CH), 128.5 (CH), 128.5 (CH), 128.2 (CH), 128.1 (CH), 128.0 (CH), 127.8 (CH), 122.0 (C-5), 103.0 (C-7), 101.5 (CH-1″), 87.5 (CH-1′), 87.4 (CH-4′), 84.1 (CH-5″), 83.0 (CH-4″), 82.3 (CH-3″), 78.1 (CH-2′), 74.9 (CH-3′), 74.4 (CH_2_), 74.4 (CH_2_), 74.2 (CH_2_), 72.7 (CH_2_), 71.0 (CH-2″), 70.3 (CH_2_-5′), 69.2 (CH_2_-6″); HRMS Calcd. For C_45_H_48_BrN_4_O_9_^+^ [M + H]^+^: 867.2599, found: 867.2599.

### 3.12. Synthesis of 5′-O-(2″,3″,4″,6″-O-Tetrabenzyl-α-d-glucopyranosyl) Tubercidin *(**16**)*

To the stirred suspension of compound **15** (200 mg, 0.23 mmol) in THF (15 mL) and methanol (15 mL) was added triethylamine (0.3 mL) and 20% Pd(OH)_2_/C (50 mg, 0.04 mmol), and the mixture was stirred at room temperature for 5 h under a continuous hydrogen environment until the reaction was completed (monitored by TLC, CH_2_Cl_2_:CH_3_OH,20:1, R_f_ 0.24). The mixture was filtered, and the filtrate was concentrated to provide the crude product, which was purified by flash column chromatography on silica gel (CH_2_Cl_2_/CH_3_OH, 20:1) to provide 151.8 mg compound **16** (83.5% yield) as a white solid. mp: 74–75 °C; ${\left[\alpha \right]}_{D}^{25}$ = 1.8 (*c* = 0.1, CH_3_OH); ^1^H NMR (400 MHz, DMSO) δ 8.06 (s, 1H, H-2), 7.63 (d, *J* = 3.6 Hz, 1H, H-8), 7.41–7.21 (m, 18H, H-Bn), 7.17–7.10 (m, 2H, H-Bn), 7.00 (s, 2H, NH_2_), 6.53 (d, *J* = 3.5 Hz, 1H, H-7), 6.16 (d, *J* = 5.9 Hz, 1H, H-1′), 5.41 (d, *J* = 6.2 Hz, 1H, 2′-OH), 5.22 (s, 1H, 3′-OH), 4.95–4.41 (m, 9H, H-1″,Bn), 4.36–4.32 (m, 1H, H-2′), 4.12–4.08 (m, 2H, H-3′,5″), 3.83–3.77 (m, 2H, H-4′,5′), 3.70–3.41 (m, 6H, H-2″,3″,4″,5′,6″); ^13^C NMR (101 MHz, DMSO): δ 157.5 (C-6), 151.7 (CH-2), 150.8 (C-4), 139.2 (CH), 138.9 (CH), 138.7 (CH), 128.7 (CH), 128.7 (CH), 128.2 (CH), 128.1 (CH), 128.1 (CH), 128.0 (CH), 127.9 (CH), 122.1 (CH-8), 103.1 (C-5), 100.4 (CH-7), 96.3 (CH-1″), 87.0 (CH-1′), 82.7 (CH-5″), 82.0 (CH-4′), 79.9 (CH-4″), 77.8 (CH-3″), 75.2 (CH_2_), 74.9 (CH-2′), 74.6 (CH_2_), 72.8 (CH_2_), 72.0 (CH_2_), 71.4 (CH-3′), 70.5 (CH-2″), 69.0 (CH_2_-6″), 68.0 (CH_2_-5′); HRMS Calcd. For C_45_H_49_N_4_O_9_^+^ [M + H]^+^: 789.3494, found: 789.3499.

### 3.13. Synthesis of 5′-O-(2″,3″,4″,6″-O-Tetrabenzyl-β-d-glucopyranosyl) Tubercidin *(**16′**)*

Compound **15′** was converted into **16′** (79.0% yield) as described for the synthesis of **16**; R_f_ 0.23 (CH_2_Cl_2_/CH_3_OH, 20:1); mp: 166–167 °C; ${\left[\alpha \right]}_{D}^{25}$ = −21 (*c* = 0.1, CH_3_OH); ^1^H NMR (400 MHz, DMSO): δ 8.03 (s, 1H, H-2), 7.38–7.18 (m, 15H, H-8, Bn), 7.19–7.05 (m, 6H, H-Bn), 6.98 (s, 2H, NH_2_), 6.59 (d, *J* = 3.6 Hz, 1H, H-7), 6.09 (d, *J* = 5.2 Hz, 1H, H-1′), 5.36 (d, *J* = 6.3 Hz, 1H, 2′-OH), 5.22 (d, *J* = 5.3 Hz, 1H, OH-3′), 4.94–4.26 (m, 10H, H-1″,2′,Bn), 4.11–4.05 (m, 3H, H-3′,5′,5″), 3.70–3.40 (m, 6H, H-2″,3″,4′,5′,6″), 3.26 (d, *J* = 8.0 Hz, 1H, H-4″); ^13^C NMR (101 MHz, DMSO): δ 157.9 (C-6), 152.3 (CH-2), 150.8 (C-4), 139.1 (CH), 138.8 (CH), 138.7 (CH), 138.6 (CH), 128.7 (CH), 128.7 (CH), 128.6 (C-Bn), 128.6 (CH), 128.5 (CH), 128.2 (CH), 128.1 (CH), 128.1 (CH), 128.1 (CH), 128.0 (CH), 127.9 (CH), 127.8 (CH), 127.8 (CH), 122.0 (CH-8), 103.3 (CH-1″), 103.2 (C-5), 100.5 (CH-7), 87.6 (CH-1′), 84.1 (CH-4′), 82.6 (CH-5″), 82.1 (CH-4″), 78.1 (CH-3″), 74.9 (CH-2′), 74.4 (CH_2_), 74.3 (CH_2_), 74.2 (CH_2_), 74.1 (CH-3′), 72.8 (CH_2_), 71.1 (CH-2″), 70.6 (CH_2_-5′), 69.1 (CH_2_-6″); HRMS Calcd. For C_45_H_49_N_4_O_9_^+^ [M + H]^+^: 789.3494, found: 789.3499.

### 3.14. Synthesis of 5′-O-α-d-Glucopyranosyl tubercidin *(**2**)*

To the stirred suspension of compound **16** (100 mg, 0.13 mmol) in THF (10 ml) and methanol (10 mL) was added 20% Pd(OH)_2_/C (50 mg, 0.04 mmol), and the mixture was stirred at 40 °C for 12 h under a continuous hydrogen environment. The mixture was filtered and the filtrate was concentrated to provide the crude product, which was purified by isocratic HPLC (H_2_O/CH_3_OH, 4:1) to provide 43.5 mg compound **2** (80.1% yield) as a white solid; mp: 120–122 °C; ${\left[\alpha \right]}_{D}^{25}$ = 10.5 (*c* = 0.08, H_2_O); ^1^H NMR (400 MHz, DMSO): δ 8.09 (s, 1H, H-2), 7.76 (d, *J* = 3.2 Hz, 1H, H-8), 7.20 (s, 2H, NH_2_), 6.61 (d, *J* = 3.4 Hz, 1H, H-7), 6.14 (d, *J* = 7.0 Hz, 1H, H-1′), 5.21 (d, *J* = 4.6 Hz, 2H, 2″, 3′-OH), 5.10 (d, *J* = 4.3 Hz, 1H, 2′-OH), 4.92 (d, *J* = 4.9 Hz, 1H, 3″-OH), 4.84 (d, *J* = 3.6 Hz, 1H, 4″-OH), 4.72 (d, *J* = 3.1 Hz, 1H, H-1″), 4.49 (s, 1H, 6″-OH), 4.43 (m, 1H, H-2′), 4.10 (s, 1H, H-4′), 4.08 (d, *J* = 1.7 Hz, 1H, H-3′), 3.78 (dd, *J* = 10.9, 2.8 Hz, 1H, H-5′), 3.66–3.63 (m, 1H, H-6′), 3.47–3.42 (m, 3H, H-5′,4″,6″), 3.40–3.34 (m, 2H, H-2″,5″), 3.13–3.07 (m, 1H, H-3″); ^13^C NMR (101 MHz, DMSO): δ 157.3 (C-6), 151.5 (CH-2), 150.6 (C-4), 122.4 (CH-8), 102.8 (C-5), 100.0 (CH-7), 98.5 (CH-1″), 85.9 (CH-1′), 83.2 (CH-4′), 74.2 (CH-2′), 73.3 (CH-4″), 72.7 (CH-5″), 71.5 (CH-2″), 70.9 (CH-3′), 70.0 (CH-3″), 67.0 (CH_2_-5′), 60.9 (CH_2_-6″); HRMS Calcd. For C_17_H_25_N_4_O_9_^+^ [M + H]^+^: 429.1616, found: 429.1620.

### 3.15. Synthesis of 5′-O-β-d-Glucopyranosyl Tubercidin *(**17**)*

Compound **16′** was converted into **17** (79.0% yield) as described for the synthesis of **2**; mp: 156–157 °C; ${\left[\alpha \right]}_{D}^{25}$ = −37 (*c* = 0.08, H_2_O); ^1^H NMR (400 MHz, DMSO): δ 8.07 (s, 1H, H-2), 7.38 (d, *J* = 3.7 Hz, 1H, H-8), 7.09 (s, 2H, 6-NH_2_), 6.61 (d, *J* = 3.6 Hz, 1H, H-7), 6.08 (d, *J* = 5.6 Hz, 1H, H-1′), 5.26 (d, *J* = 5.5 Hz, 1H, 2′-OH), 5.17 (d, *J* = 4.6 Hz, 1H, 3′-OH), 5.03 (d, *J* = 3.7 Hz, 1H, 2″-OH), 4.95 (s, 1H, 3″-OH), 4.95 (s, 1H, 4″-OH), 4.51 (s, 1H, 6″-OH), 4.36 (d, *J* = 5.0 Hz, 1H, H-2′), 4.21 (d, *J* = 7.8 Hz, 1H, H-1″), 4.14 (d, *J* = 4.4 Hz, 1H, H-3′), 4.02–3.95 (m, 2H, H-4′,5′), 3.68 (d, *J* = 9.2 Hz, 1H, H-6″), 3.59 (dd, *J* = 11.9, 5.7 Hz, 1H, H-5′), 3.44 (d, *J* = 6.7 Hz, 1H, H-6″), 3.15 (d, *J* = 9.9 Hz, 1H, H-3″), 3.12–3.04 (m, 2H, H-4″,5″), 3.00 (d, *J* = 7.8 Hz, 1H, H-2″); ^13^C NMR (101 MHz, DMSO): δ 157.5 (C-6), 151.7 (CH-2), 150.7 (C-4), 122.2 (CH-8), 103.5 (CH-1″), 103.1 (C-5), 100.5 (CH-7), 87.1 (CH-1′), 83.1 (CH-4′), 77.4 (CH-2″), 77.2 (CH-2′), 74.2 (CH-4″), 74.1 (C-5″), 71.1 (CH-3′), 70.5 (CH-3″), 69.5 (CH_2_-5′), 61.5 (CH_2_-6″); HRMS Calcd. For C_17_H_25_N_4_O_9_^+^ [M + H]^+^: 429.1616, found: 429.1619.

## 4. Conclusions

In summary, we developed a concise total synthesis of 5′-*O*-α-d-glucopyranosyl tubercidin **2** in eight steps with a 28% overall yield from commercially available 1-*O*-acetyl-2,3,5-tri-*O*-benzoyl-β-d-ribose **7**. This is the first report on the synthesis of disaccharide 7-deazapurine nucleosides. This synthetic route features two key steps: (1) a one-pot Vorbrüggen glycosylation of ribose **7** with 6-chloro-7-bromo-7-deazapurine **8**, and (2) stereoselective α-*O*-glycosylation of 7-deazapurine nucleoside **11** with 2,3,4,6-tetra-*O*-benzyl-glucopyranosyl trichloroacetimidate **6**. Additionally, 5′-*O*-β-d-glucopyranosyl tubercidin **17** was also prepared. A comparison with the natural product’s spectra further confirmed the reported structure. Applications of this newly developed modular synthetic approach to prepare other glycosylated tubercidin analogs are ongoing in our laboratory.

## Supplementary Materials

The following are available online at https://www.mdpi.com/1660-3397/18/8/398/s1, Table S1: ^13^C NMR chemical shifts of naturally occurring nucleoside **2** and synthetic nucleosides **2** and **17**; Table S2: ^1^H NMR chemical shifts and coupling constants of naturally occurring nucleoside **2**, synthetic **2** and **17**, and ^1^H-and ^13^C-NMR charts of all compounds.
