# Supplementary material for: First Total Synthesis of 5′-O-α-d-Glucopyranosyl Tubercidin
Source: Mar Drugs. 2020 Jul 29;18(8):398. doi: 10.3390/md18080398 (PMC7459636; doi:10.3390/md18080398)
Supplement: Supplementary file 1 [file marinedrugs-18-00398-s001.pdf]

Supplementary material for First Total Synthesis of 5'-*O*-α-D-Glucopyranosyl Tubercidin

Wenliang Ouyang, Haiyang Huang*, Ruchun Yang, Haixin Ding, Qiang Xiao*

Jiangxi Key Laboratory of Organic Chemistry, Institute of Organic Chemistry, Jiangxi Science & Technology Normal University, Nanchang, 330013, China.

*Correspondence: huanghaiyang1209@163.com (H.H.), xiaoqiang@tsinghua.org.cn (Q.X.)

Table of Contents

1. Chemical shifts of naturally occurring nucleoside **2**, Synthetic nucleoside **2** and **17** s2

Table S1. ^{13}C NMR chemical shifts of naturally occurring nucleoside **2**, synthetic nucleoside **2** and **17** s2

Table S2. ^{1}H NMR chemical shifts and coupling constants of naturally occurring nucleoside **2**, synthetic **2** and **17** s3

2. Optimization of glycosylation of **11** and **6**........s4

3. ^{1}H and ^{13}C NMR spectra of compounds **2** – **17**........s5-s20

1. Chemical shifts of naturally occurring nucleoside **2**, Synthetic nucleoside **2** and **17**

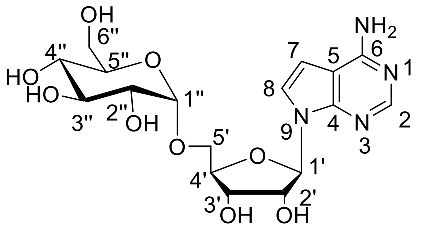

2

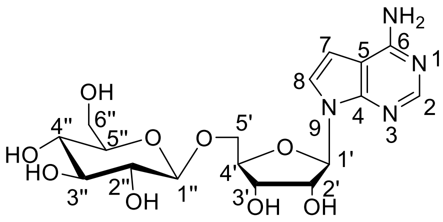

17

Table S1. ^{13}C NMR chemical shifts of naturally occurring nucleoside **2**, synthetic nucleoside **2** and **17**

Chemical shifts (δ) Position of carbon	Naturally occurring nucleoside **2** / ppm	Synthetic nucleoside **2** / ppm	Synthetic nucleoside **17** / ppm
C-6	157.3	157.3	157.5
CH-2	151.5	151.5	151.7
C-4	150.6	150.6	150.7
CH-8	122.2	122.4	122.2
C-5	103.0	102.8	103.1
CH-7	99.8	100.0	100.5
CH-1″	98.7	98.5	103.5
CH-1′	85.8	85.9	87.1
CH-4′	83.0	83.2	83.1
CH-2′	74.0	74.2	77.2
CH-4″	73.4	73.3	74.2
CH-5″	72.9	72.7	74.1
CH-2″	71.8	71.5	77.4
CH-3′	71.1	70.9	71.1
CH-3″	70.2	70.0	70.5
CH_2-5′	67.2	67.0	69.5
CH_2-6″	60.9	60.9	61.5

Table S2. ^{1}H NMR chemical shifts and coupling constants of naturally occurring nucleoside 2, synthetic 2 and 17

Chemical shifts (δ) Hydrogen	Naturally occurring nucleoside **2** (coupling constants) / ppm	Synthetic nucleoside **2** (coupling constants) / ppm	Synthetic nucleoside **17** (coupling constants) / ppm
H-2	8.03 (s)	8.09 (s)	8.07 (s)
H-8	7.69(d, *J* = 3.7 Hz)	7.76 (d, *J* = 3.2 Hz)	7.38 (d, *J* = 3.7 Hz)
NH_2	6.95 (s)	7.20 (s)	7.09 (s)
H-7	6.53 (d, *J* = 3.7 Hz)	6.61 (d, *J* = 3.4 Hz)	6.61 (d, *J* = 3.6 Hz)
H-1′	6.12(d, *J* = 7.0 Hz)	6.14 (d, *J* = 7.0 Hz)	6.08 (d, *J* = 5.6 Hz)
3′-OH	5.19 (d, *J* = 4.8 Hz)	5.21 (d, *J* = 4.6 Hz)	5.17 (d, *J* = 4.6 Hz)
2″-OH	5.19 (d, *J* = 4.8 Hz)	5.21 (d, *J* = 4.6 Hz)	5.03 (d, *J* = 3.7 Hz)
2′-OH	5.10 (d, *J* = 4.8 Hz)	5.10 (d, *J* = 4.3 Hz)	5.26 (d, *J* = 5.5 Hz)
3″-OH	4.93 (d, *J* = 3.8 Hz)	4.92 (d, *J* = 4.9 Hz)	4.95 (s)
4″-OH	4.85 (d, *J* = 4.6 Hz)	4.84 (d, *J* = 3.6 Hz)	4.91 (s)
H-1″	4.70 (d, *J* = 3.5 Hz)	4.72 (d, *J* = 3.1 Hz)	4.21 (d, *J* = 7.8 Hz)
6″-OH	4.50 (d, *J* = 4.7 Hz)	4.49 (m)	4.51 (s)
H-2′	4.41 (td, *J* =6.3 Hz, 4.8,4.8 Hz)	4.43 (m)	4.36 (d, *J* = 5.0 Hz)
H-4′	4.09 (td, *J* =4.9 Hz, 4.9,2.1 Hz)	4.11 (m)	4.02–3.95 (m)
H-3′	4.05 (m)	4.08 (d, *J* = 1.7 Hz)	4.14 (d, *J* = 4.4 Hz
H-5′	3.76 (dd, *J* = 11.1,3.1 Hz)	3.78 (dd, *J* = 10.9, 2.8 Hz)	4.02–3.95 (m)
H-6″	3.64 (ddd, *J* = 10.9,4.8,1.2 Hz)	3.66-3.63 (m)	3.68 (d, *J* = 9.2 Hz)
H-4″	3.45 (m)	3.47-3.42 (m)	3.12–3.04 (m)
H-6′ ″	3.44 (m)	3.47-3.42 (m)	3.44 (d, *J* = 6.7 Hz)
H-5′	3.41 (dd, *J* =11.1, 4.9 Hz)	3.47-3.42 (m)	3.59 (dd, *J* =11.9,5.7 Hz)
H-5″	3.34 (m)	3.40-3.34 (m)	3.12–3.04 (m)
H-2″	3.26 (m)	3.40-3.34 (m)	3.00 (d, *J* = 7.8 Hz)
H-3″	3.10 (td, *J* = 9.3, 3.8 Hz)	3.13-3.07 (m)	3.15 (d, *J* = 9.9 Hz)

2. Optimization of glycosylation of **11** and **6**

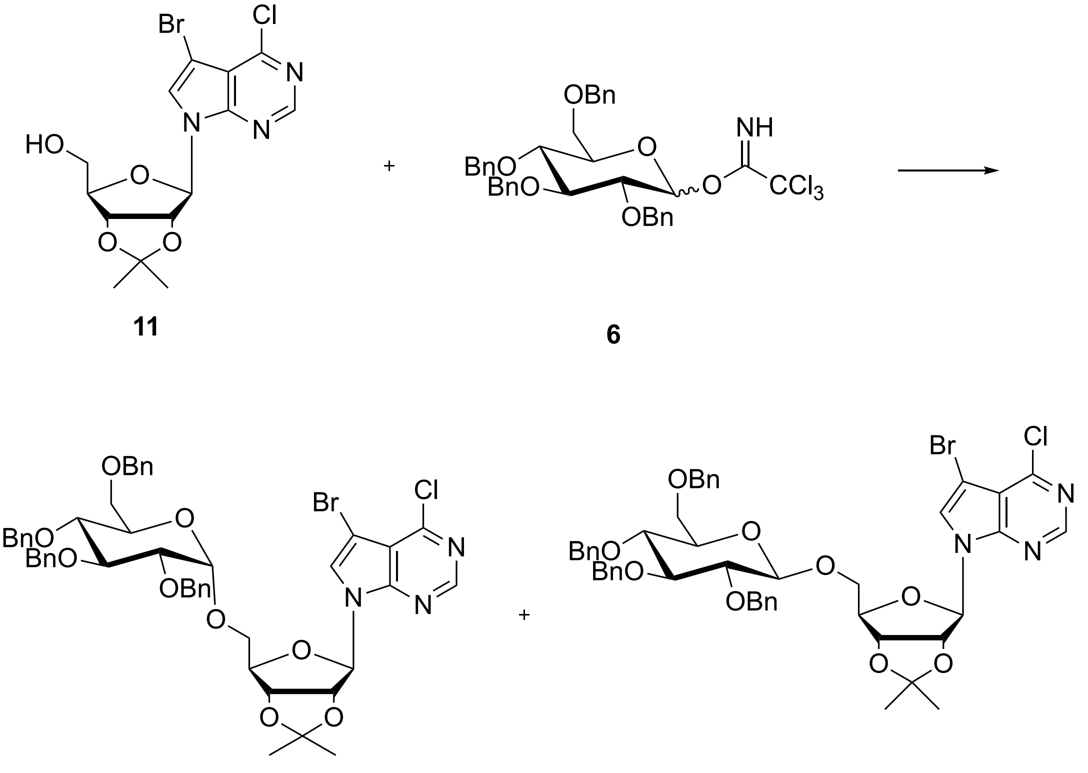

Table S3 Optimization of glycosylation of **11** and **6**

Entry	Solvent	Lewis Acid	Temperature	Ratio of 12:13	Yield
1	DCM	TMSOTf	-78 °C	2.6:1	71%
2	DCM	TMSOTf	-30 °C	4:1	79%
3	DCM	TMSOTf	0 °C	1.6:1	67%
4	DCM	$BF_3.Et_2O$	0 °C	1.2:1	43%
5	DCM	$BF_3.Et_2O$	-30 °C	3:1	59%
6	DCM	$BF_3.Et_2O$	-78 °C	1.5:1	57%

3. ^{1}H and ^{13}C NMR spectra of compounds **2 – 17**.

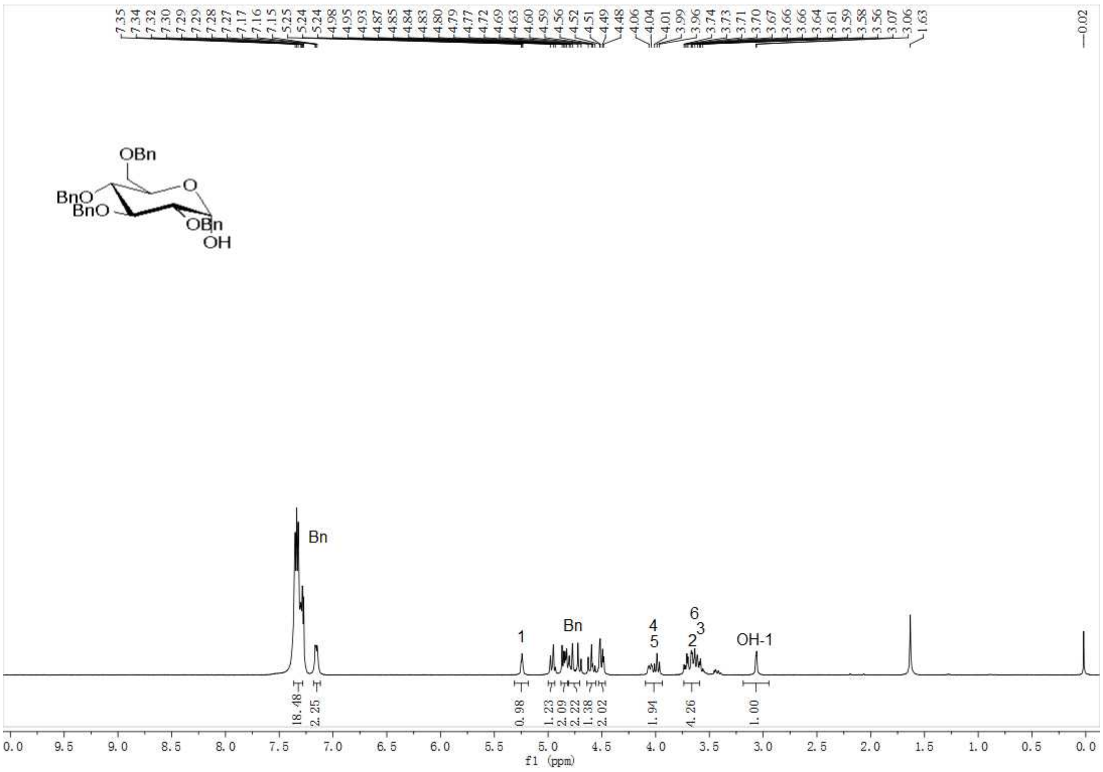

^{1}H NMR ($CDCl_3$) of 2,3,4,6-*O*-tetrabenzyl-D-glucopyranose.

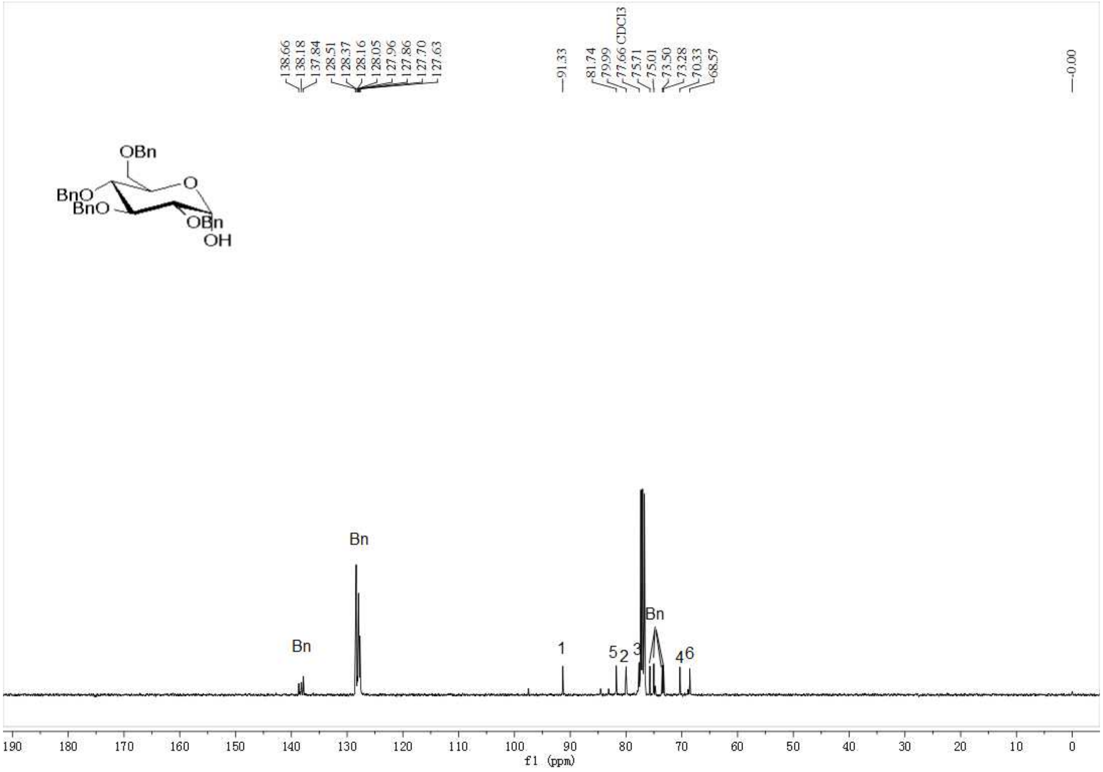

^{13}C NMR ($CDCl_3$) of 2,3,4,6-*O*-tetrabenzyl-D-glucopyranose.

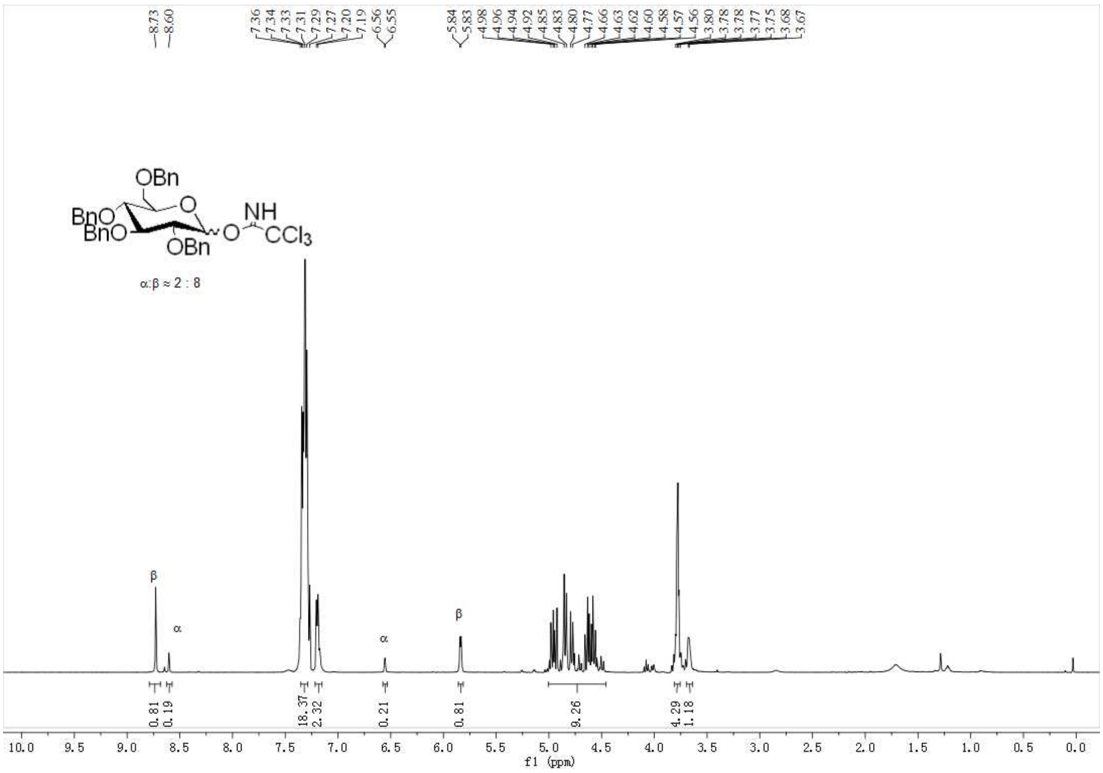

^{1}H NMR ($CDCl_3$) of **6.**

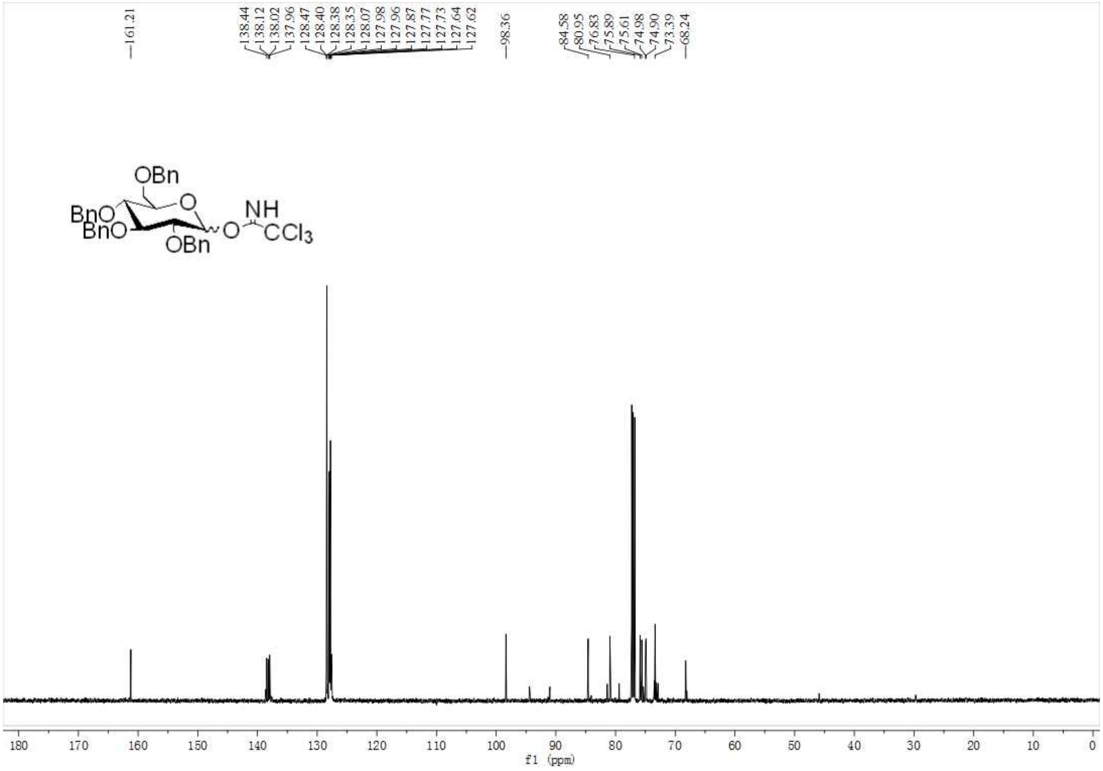

^{13}C NMR ($CDCl_3$) of **6**.

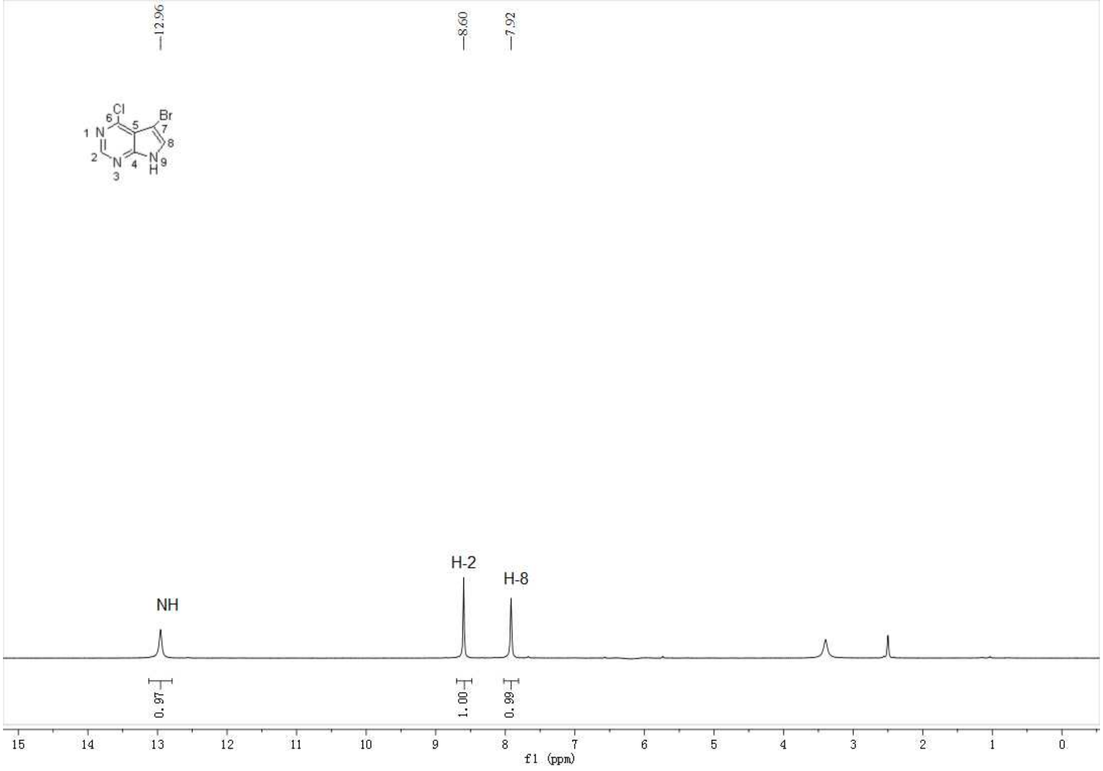

^{1}H NMR (DMSO) of **8**.

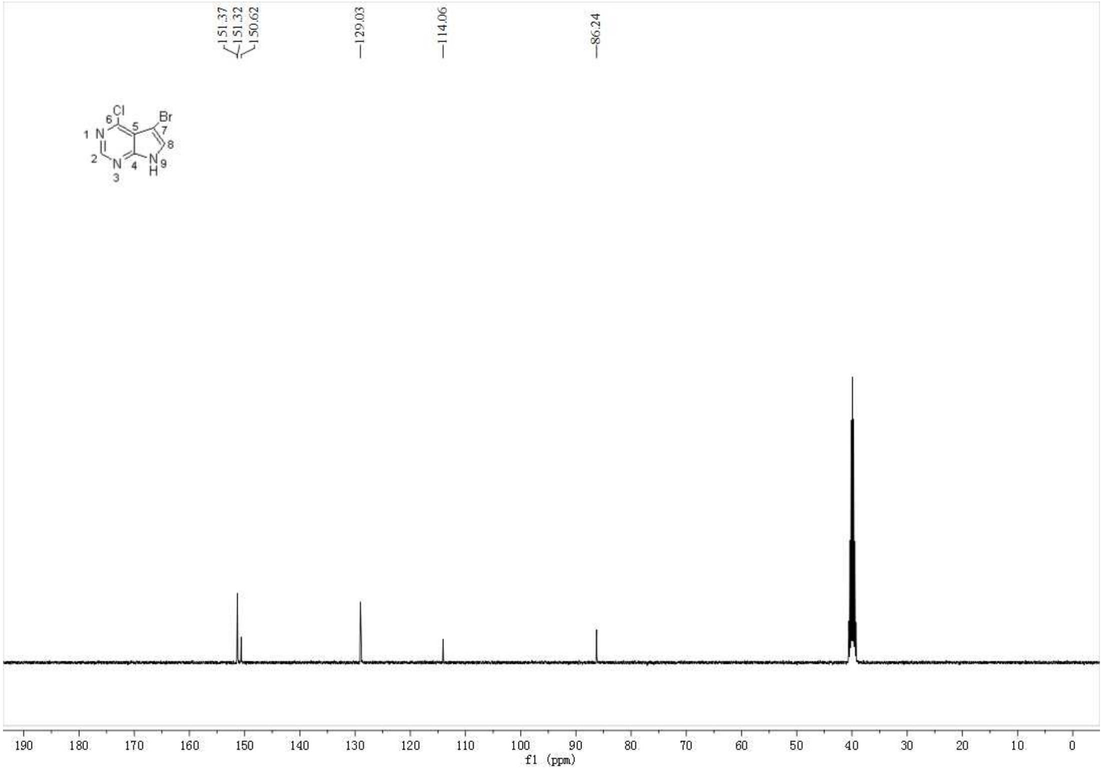

^{13}C NMR (DMSO) of **8**.

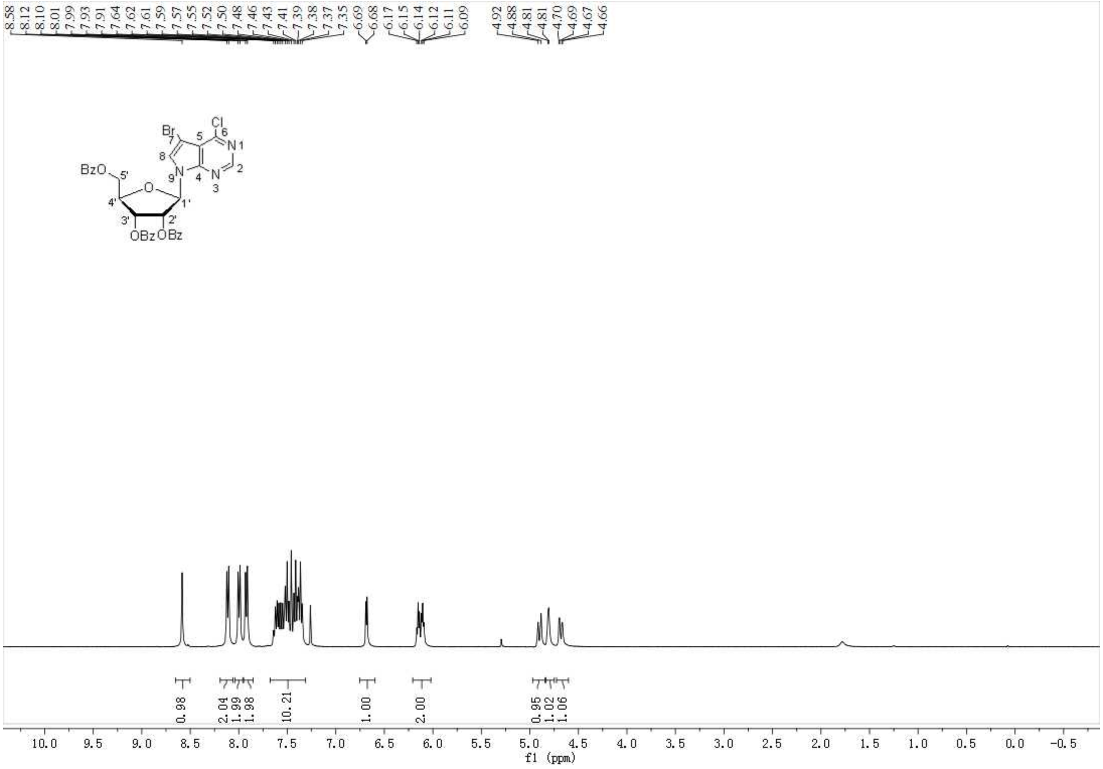

^{1}H NMR ($CDCl_3$) of **9**.

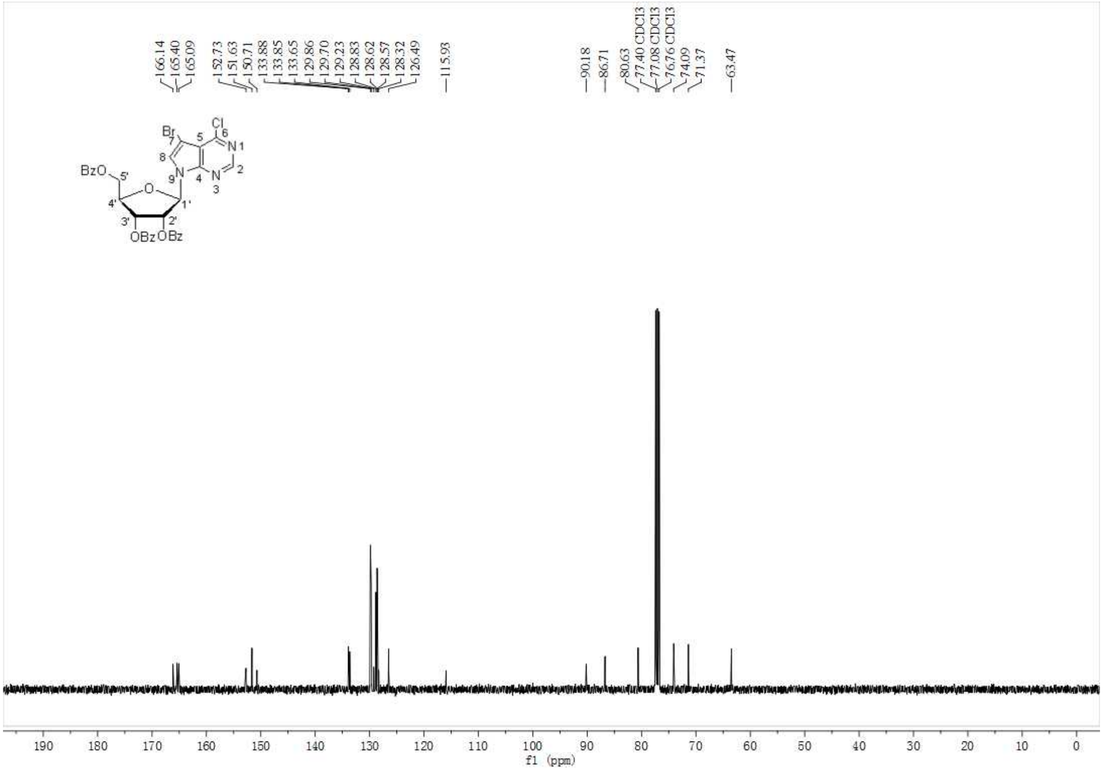

^{13}C NMR ($CDCl_3$) of **9**.

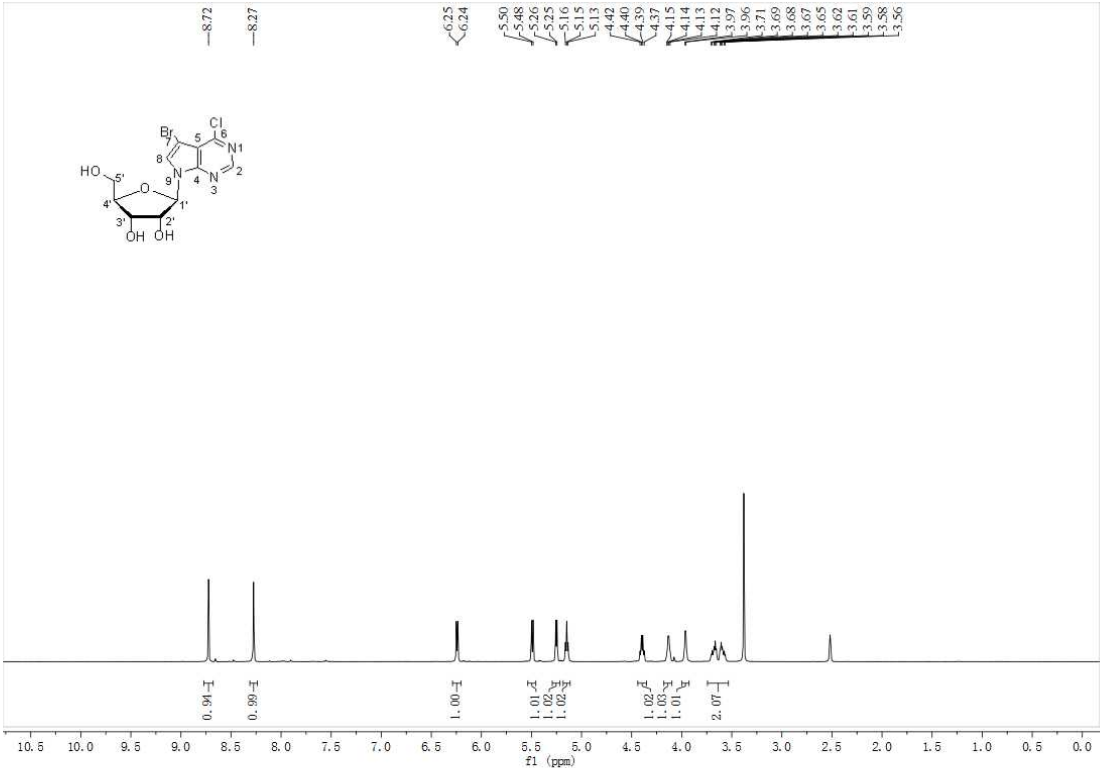

^{1}H NMR (DMSO) of **10**.

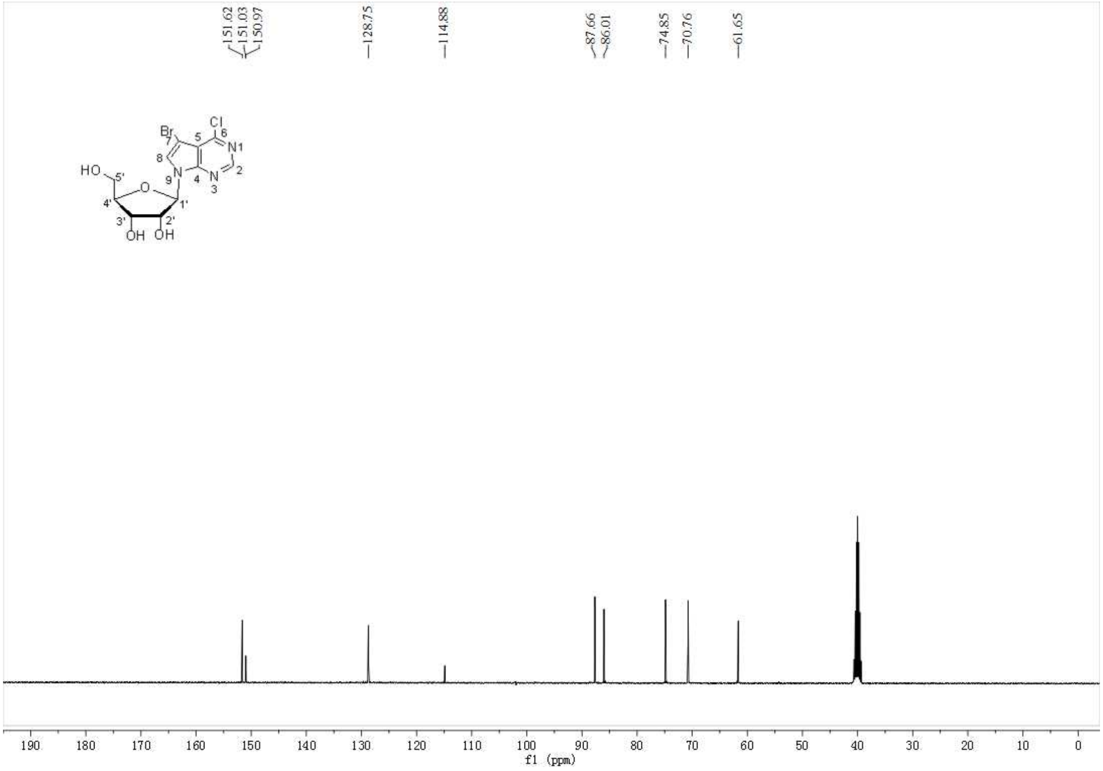

^{13}C NMR (DMSO) of **10**.

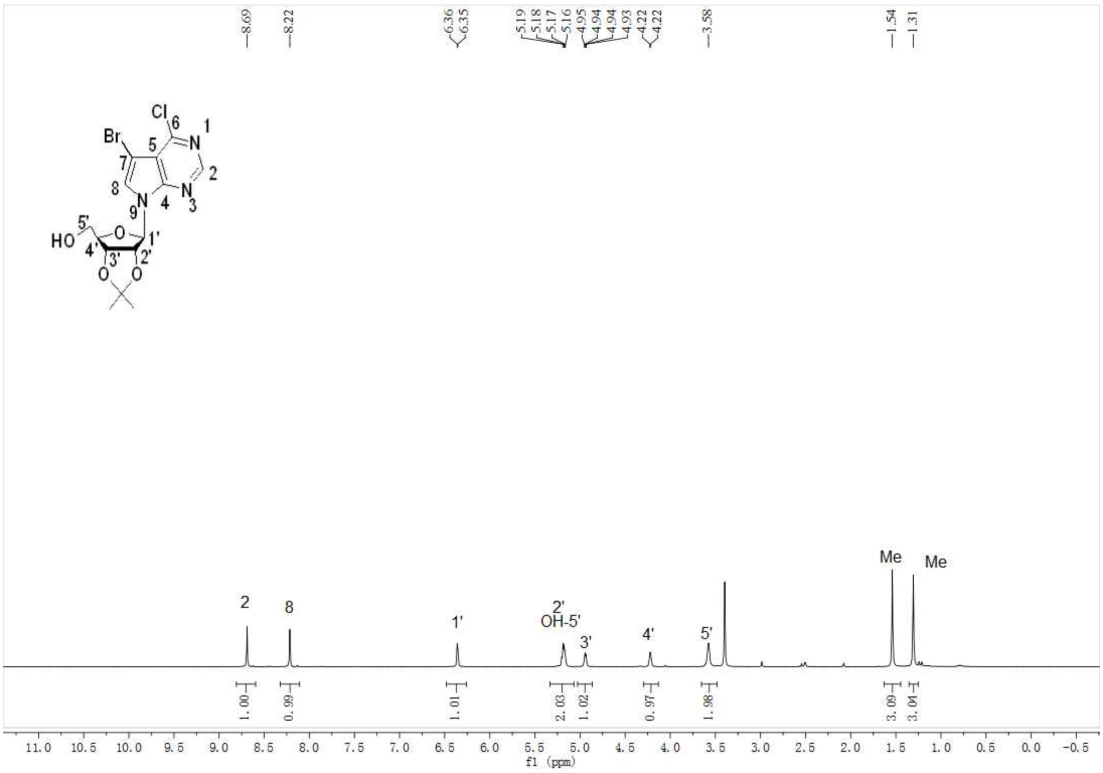

^{1}H NMR (DMSO) of **11**.

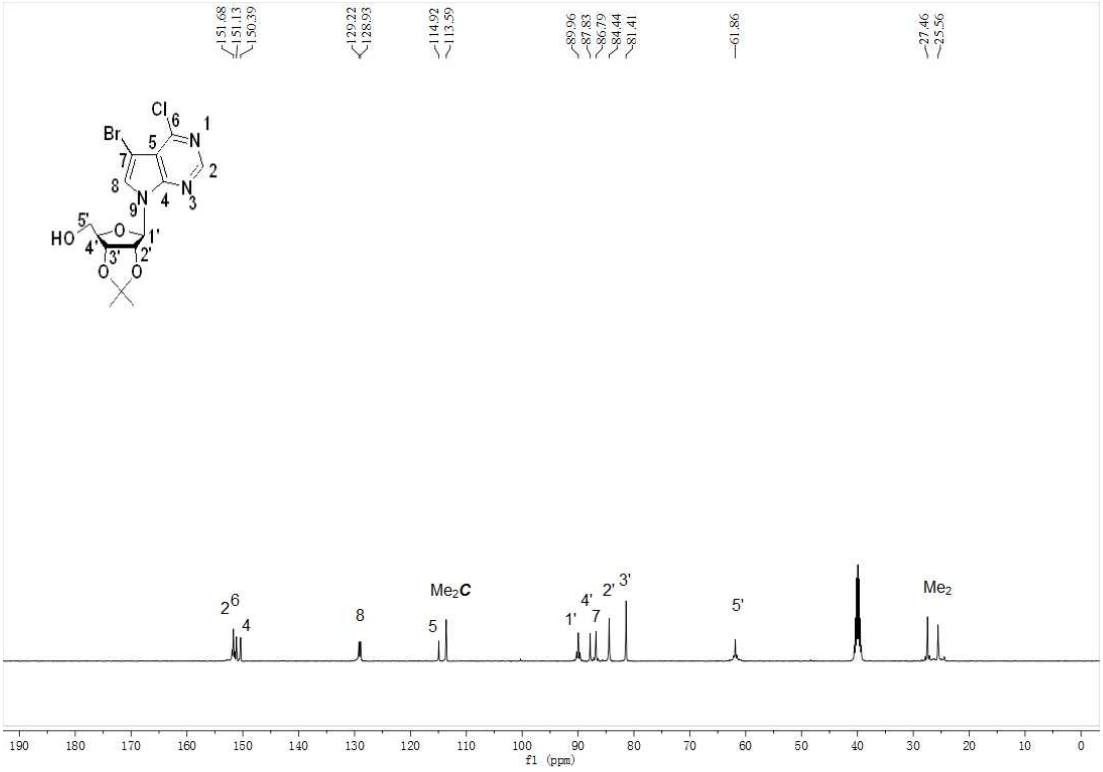

^{13}C NMR (DMSO) of **11**.

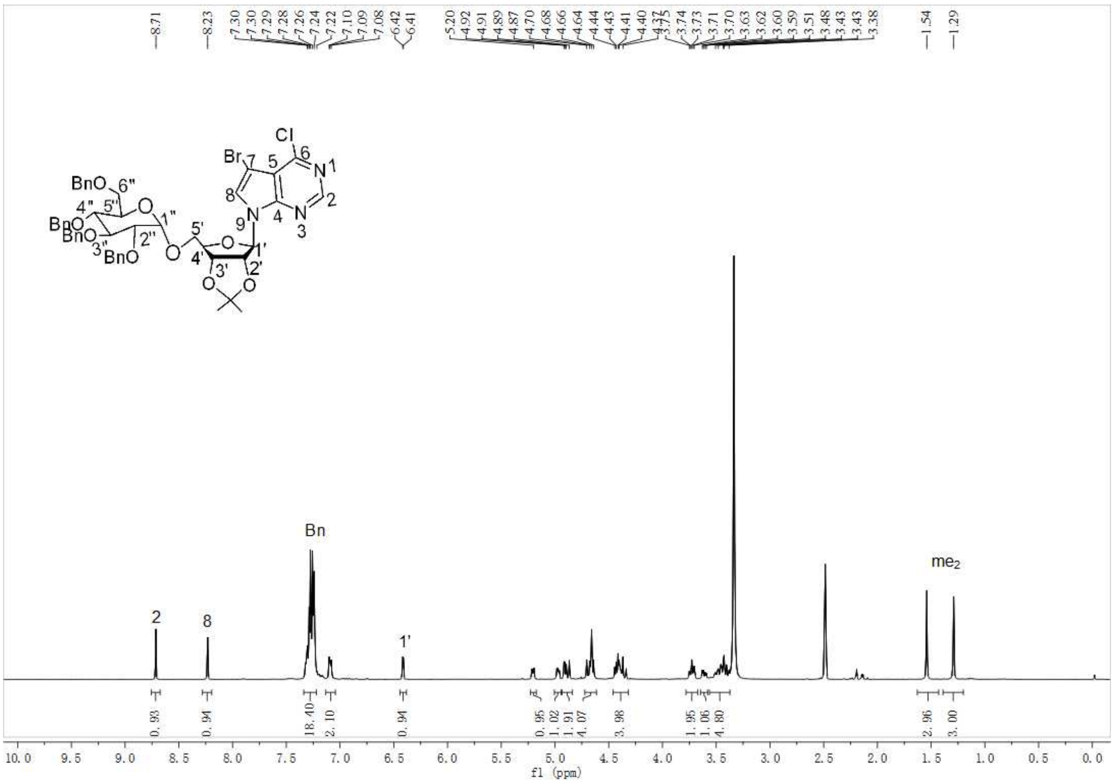

^{1}H NMR (DMSO) of **12**.

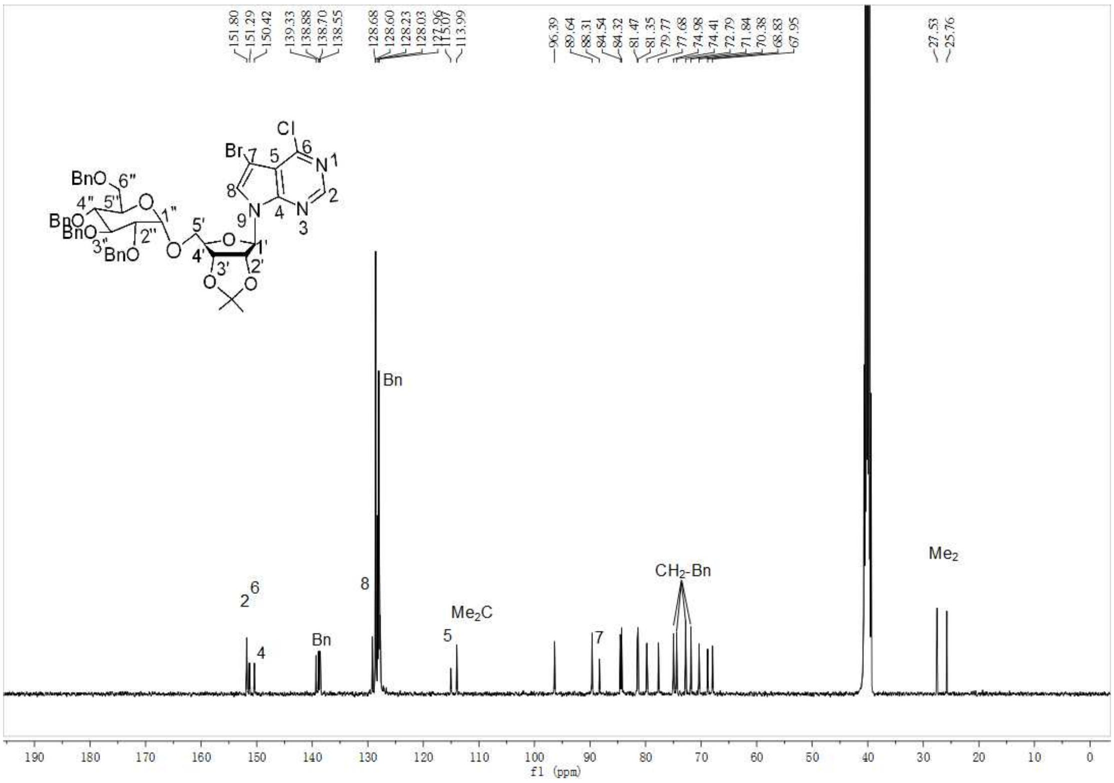

^{13}C NMR (DMSO) of **12**.

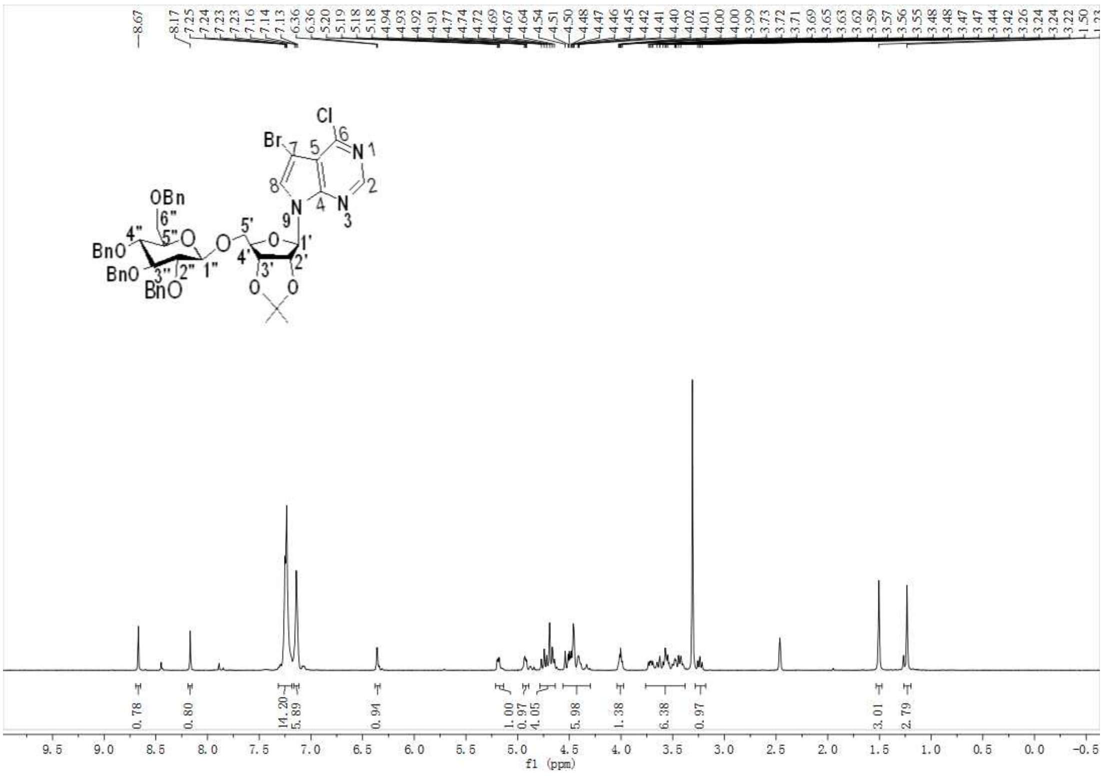

^{1}H NMR (DMSO) of **13**.

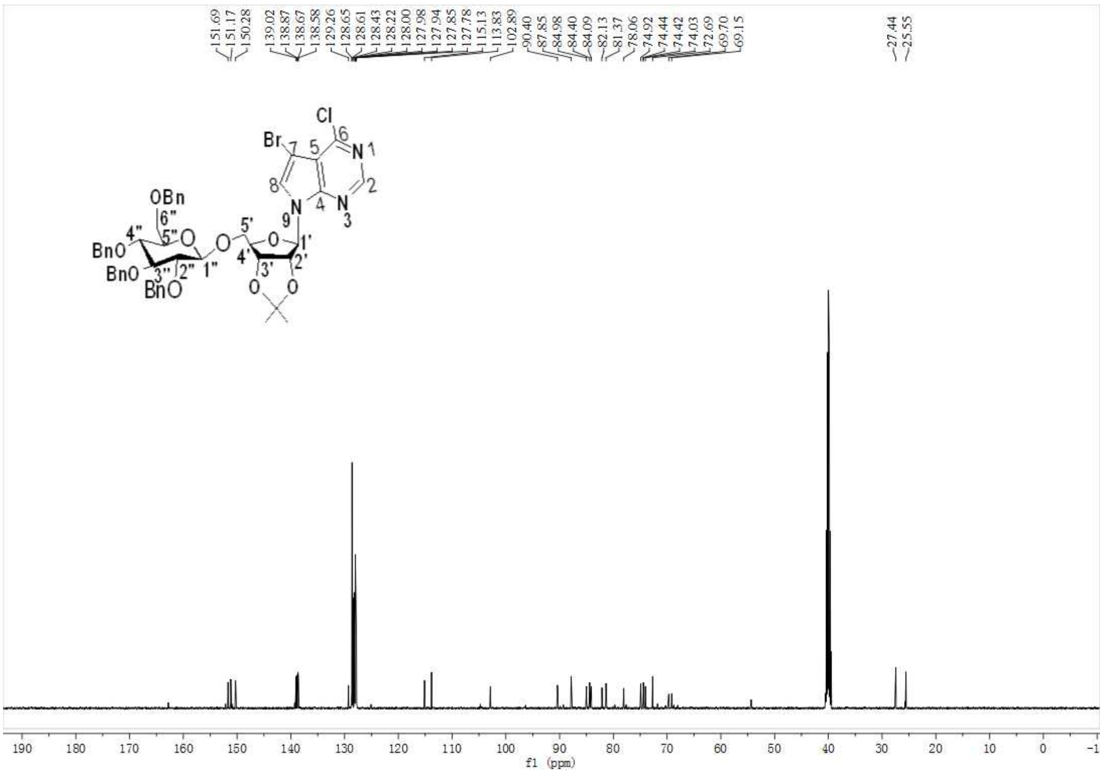

^{13}C NMR (DMSO) of **13**.

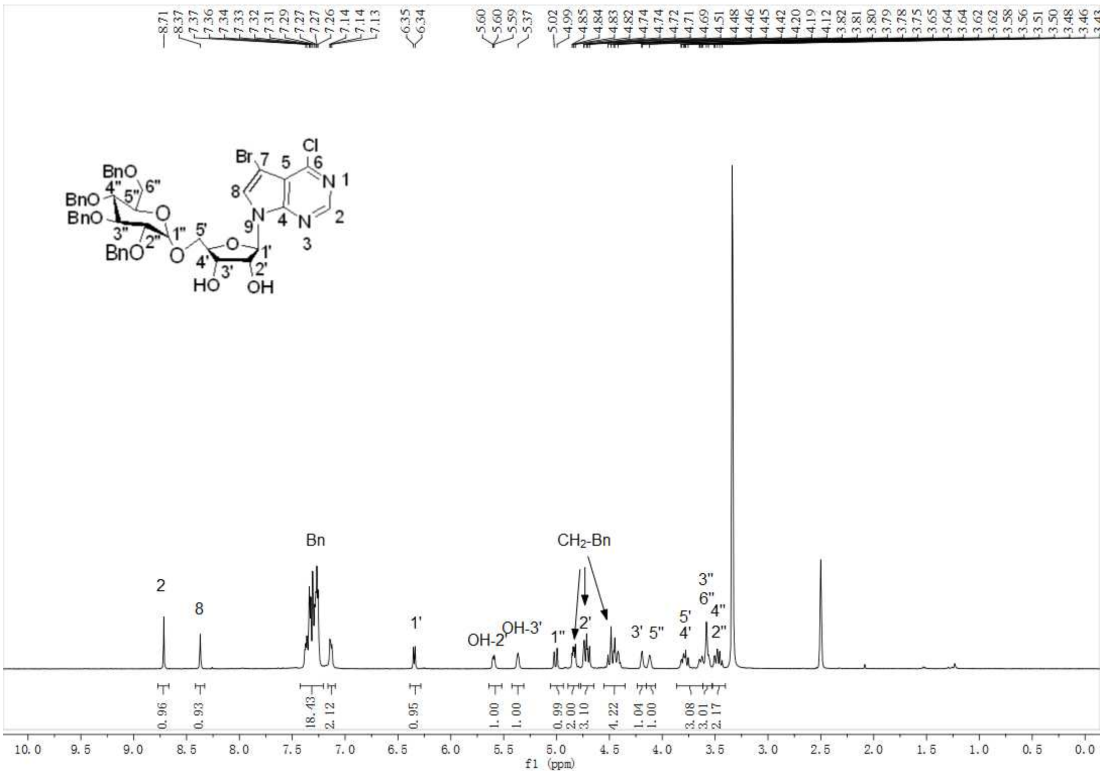

^{1}H NMR (DMSO) of **14**.

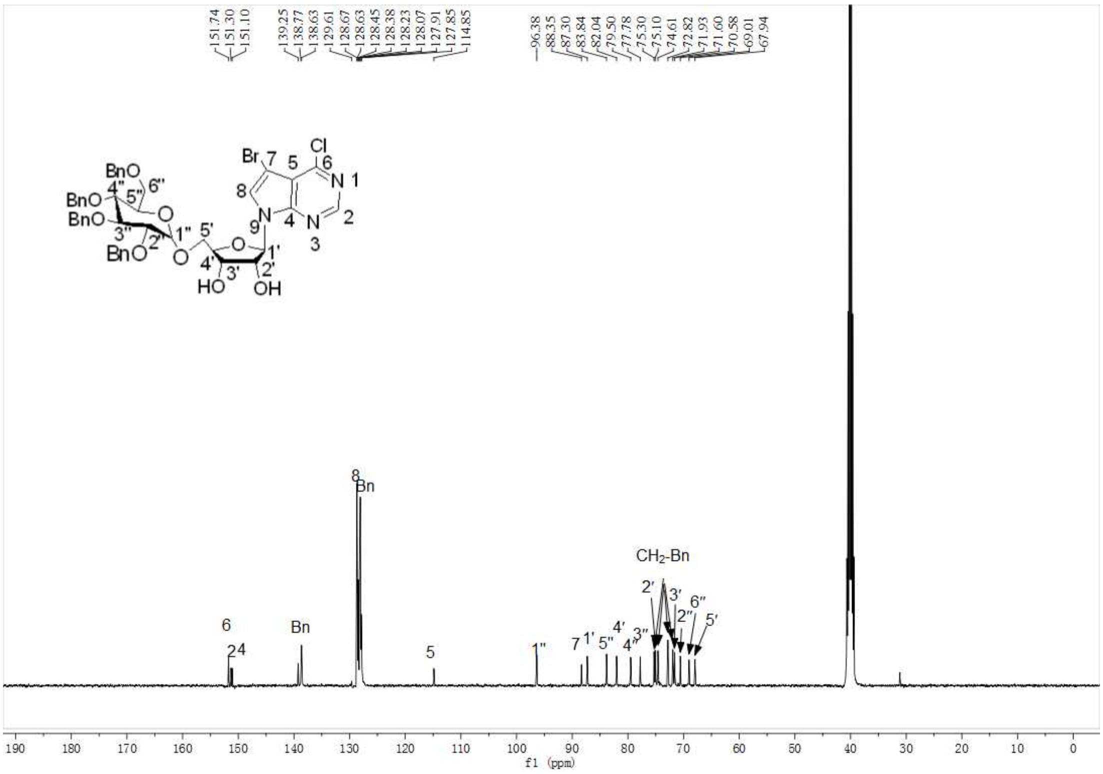

^{13}C NMR (DMSO) of **14**.

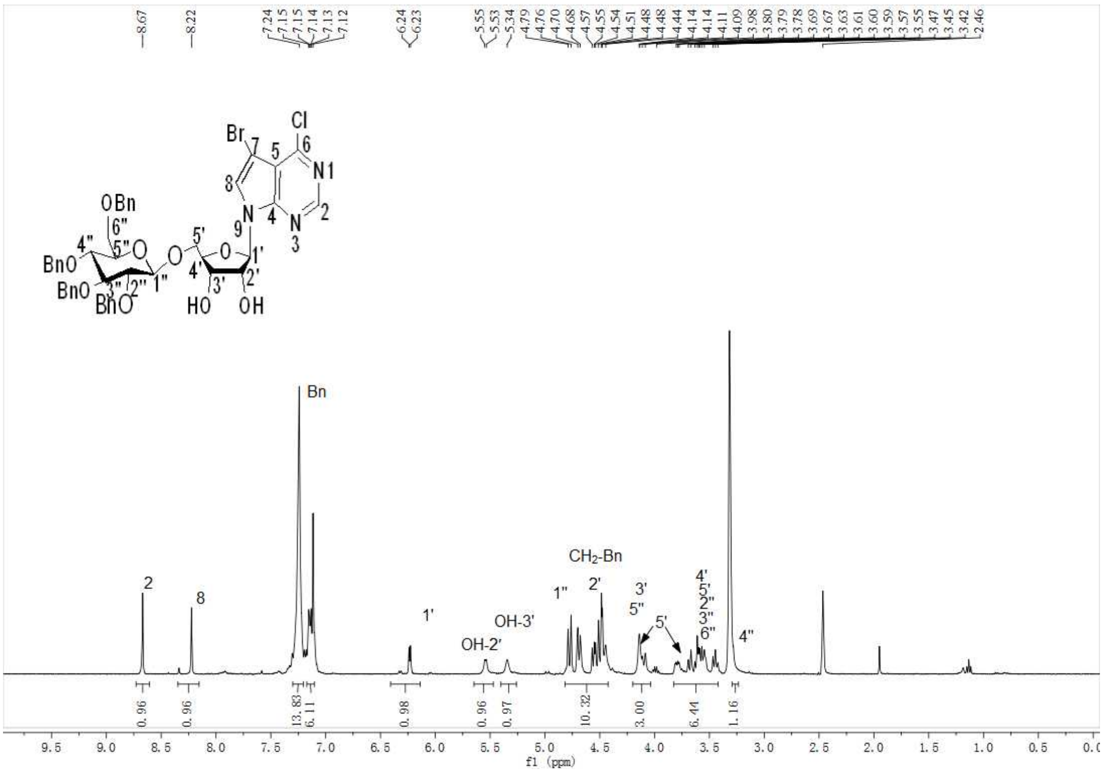

^{1}H NMR (DMSO) of **14′**.

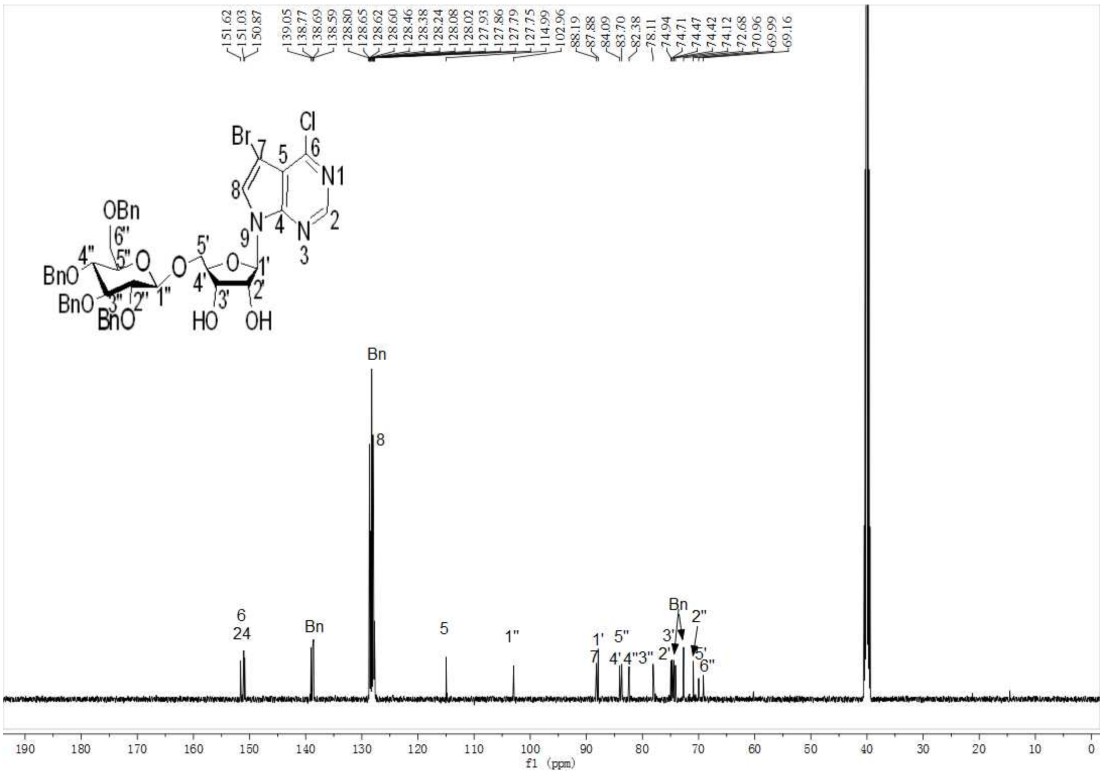

^{13}C NMR (DMSO) of **14′**.

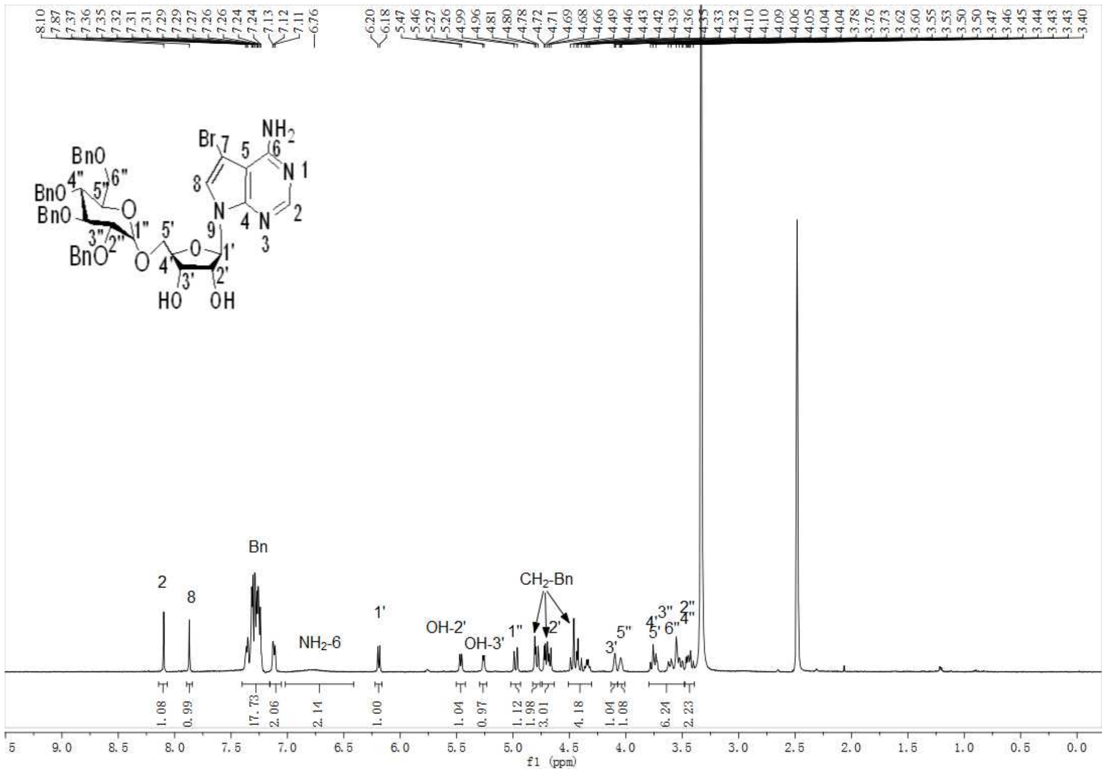

^{1}H NMR (DMSO) of **15**.

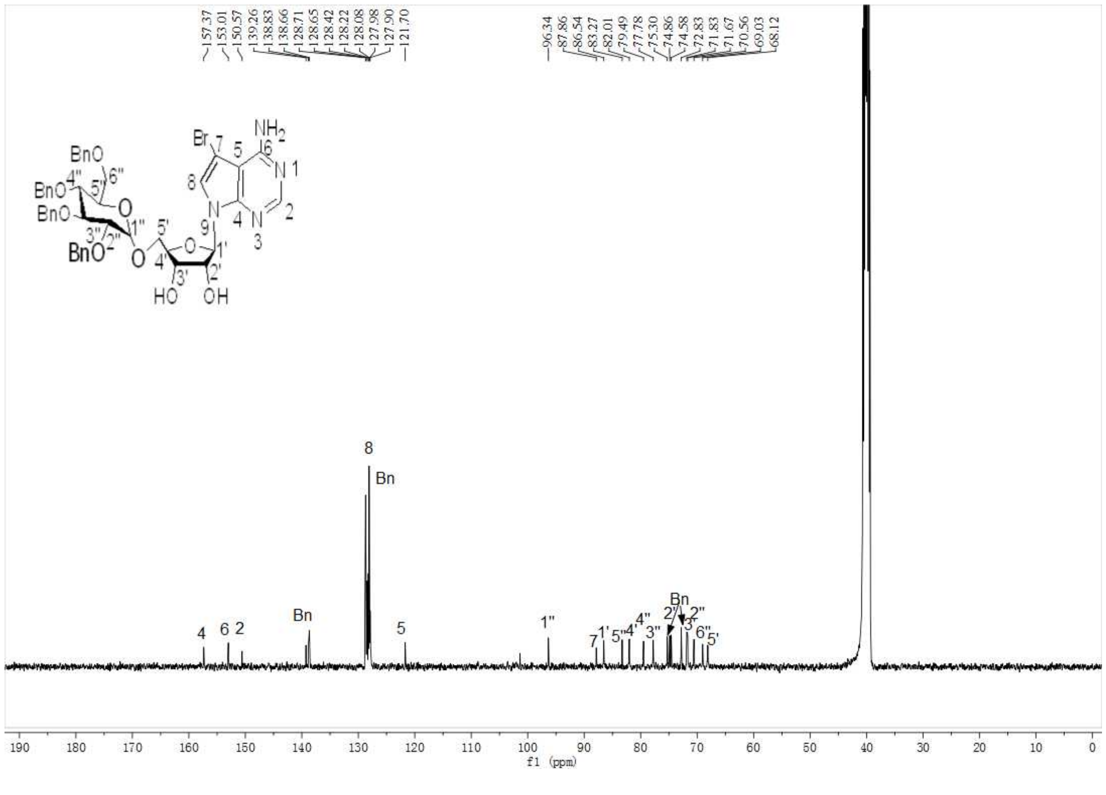

^{13}C NMR (DMSO) of **15**.

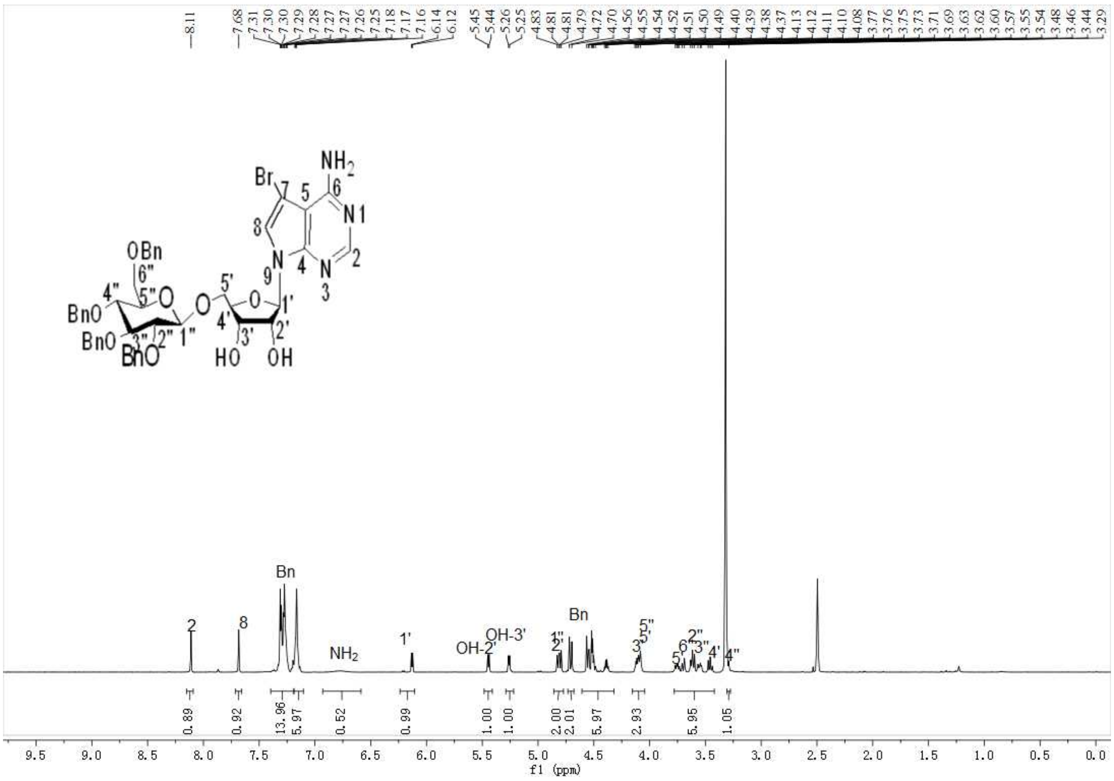

^{1}H NMR (DMSO) of **15′**.

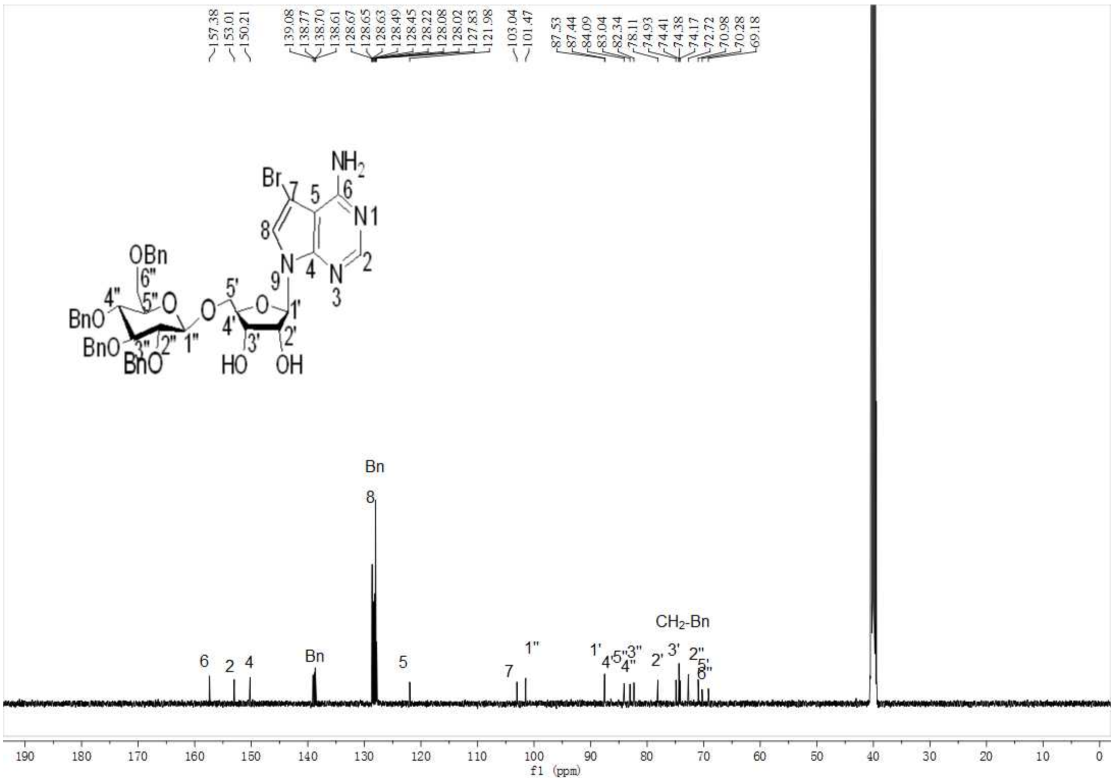

^{13}C NMR (DMSO) of **15′**.

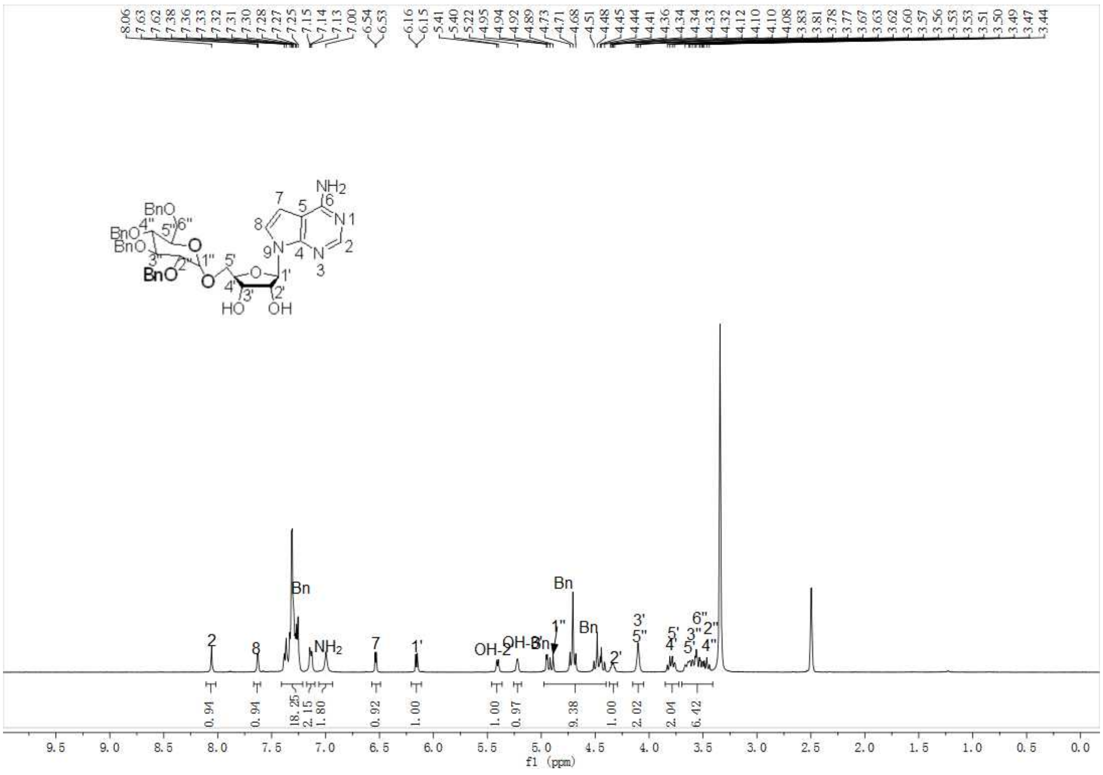

^{1}H NMR (DMSO) of **16**.

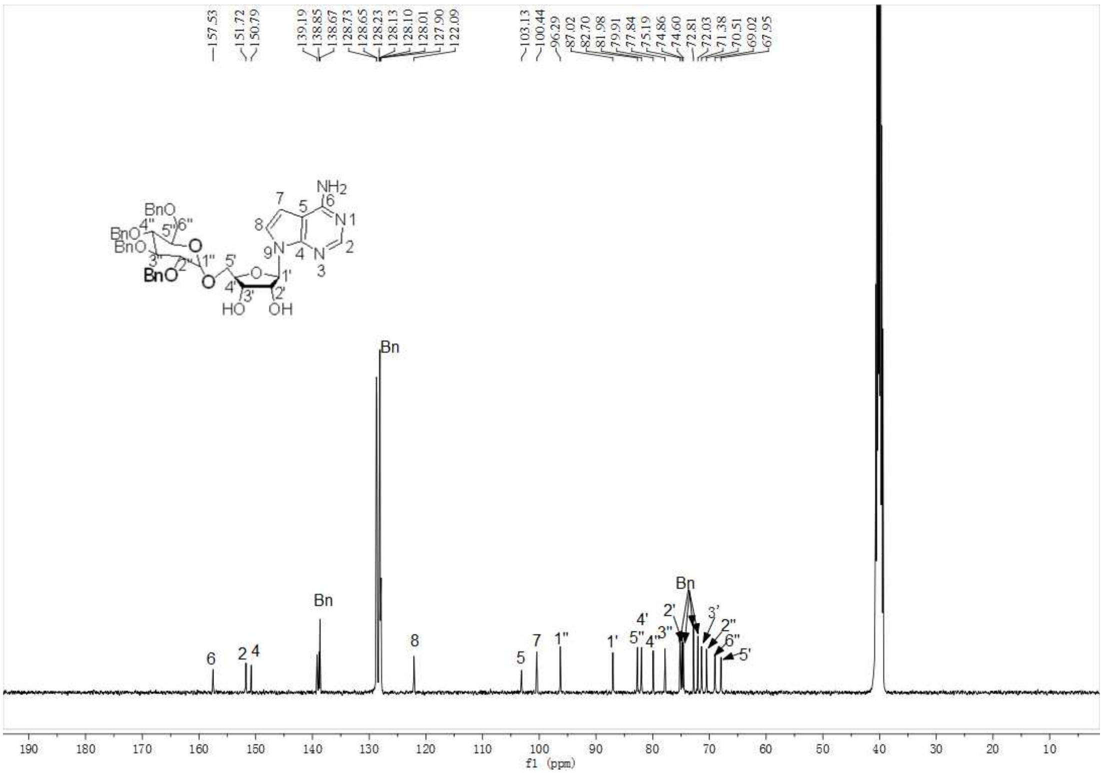

^{13}C NMR (DMSO) of **16**.

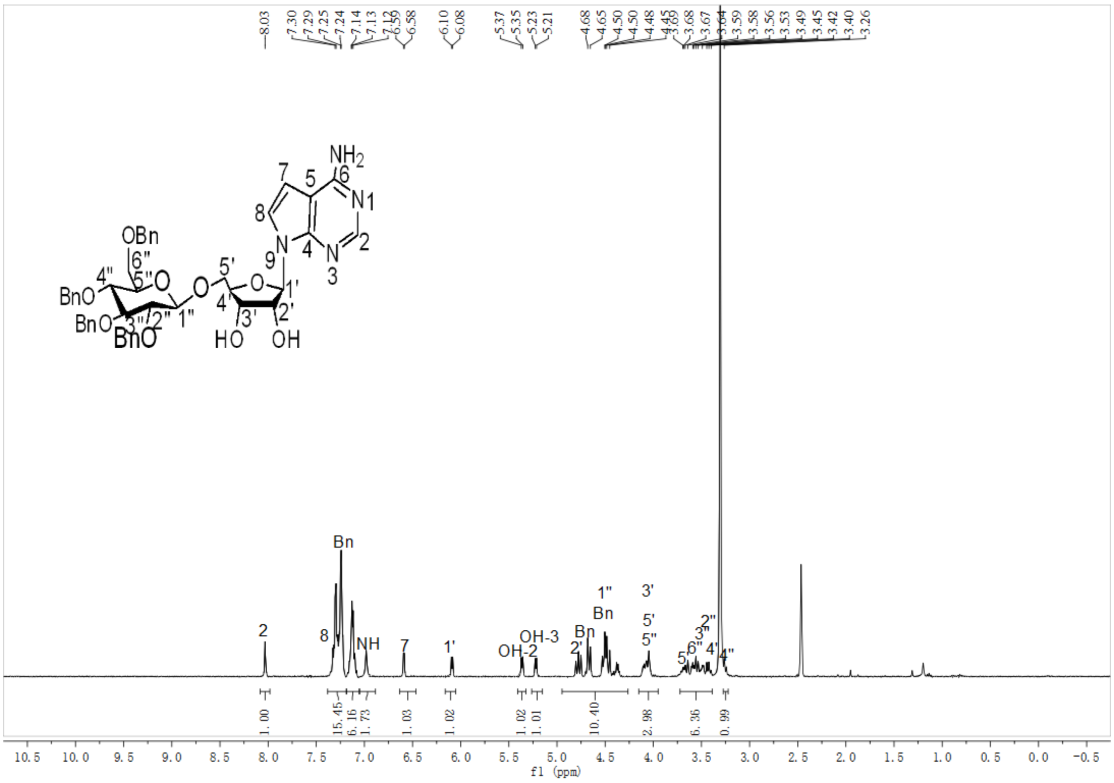

^{1}H NMR (DMSO) of **16′**.

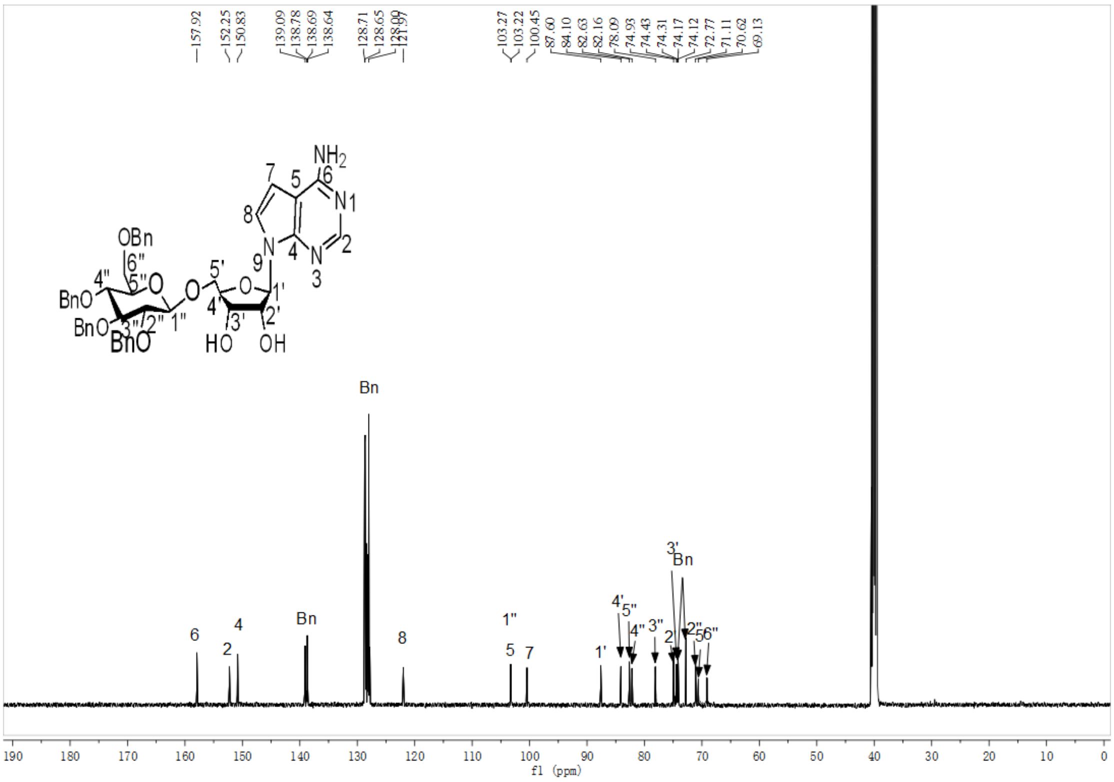

^{13}C NMR (DMSO) of **16′**.

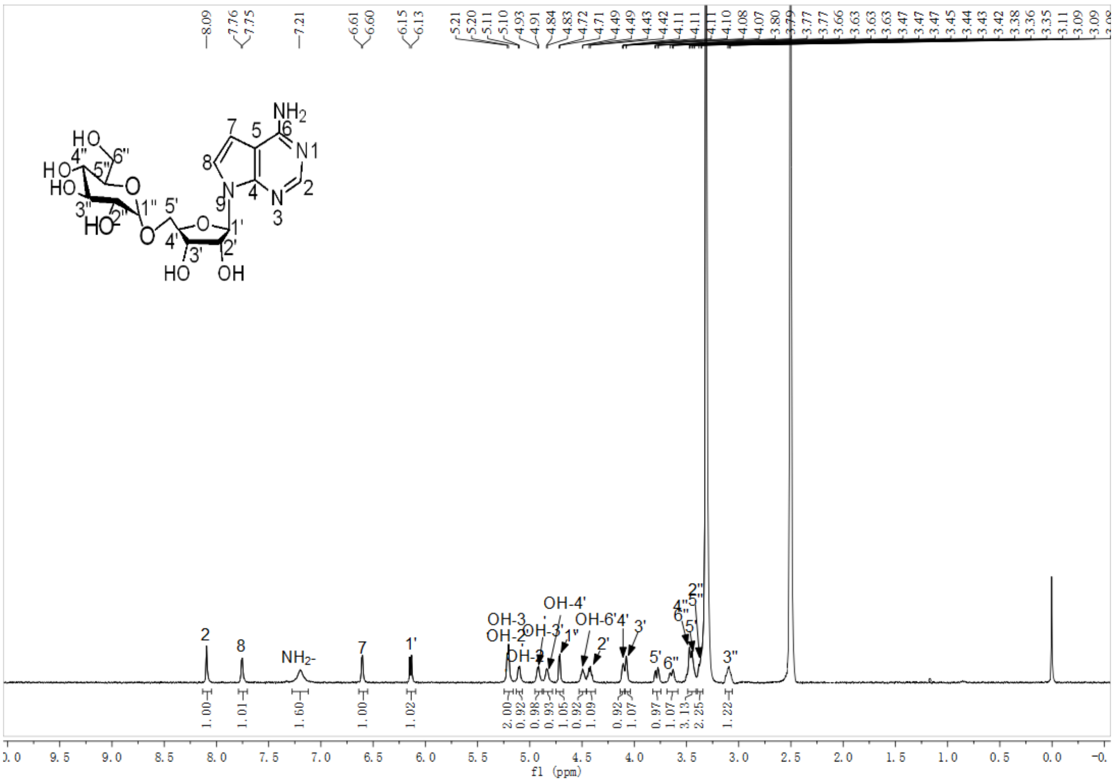

^{1}H NMR (DMSO-*d*) of **2**.

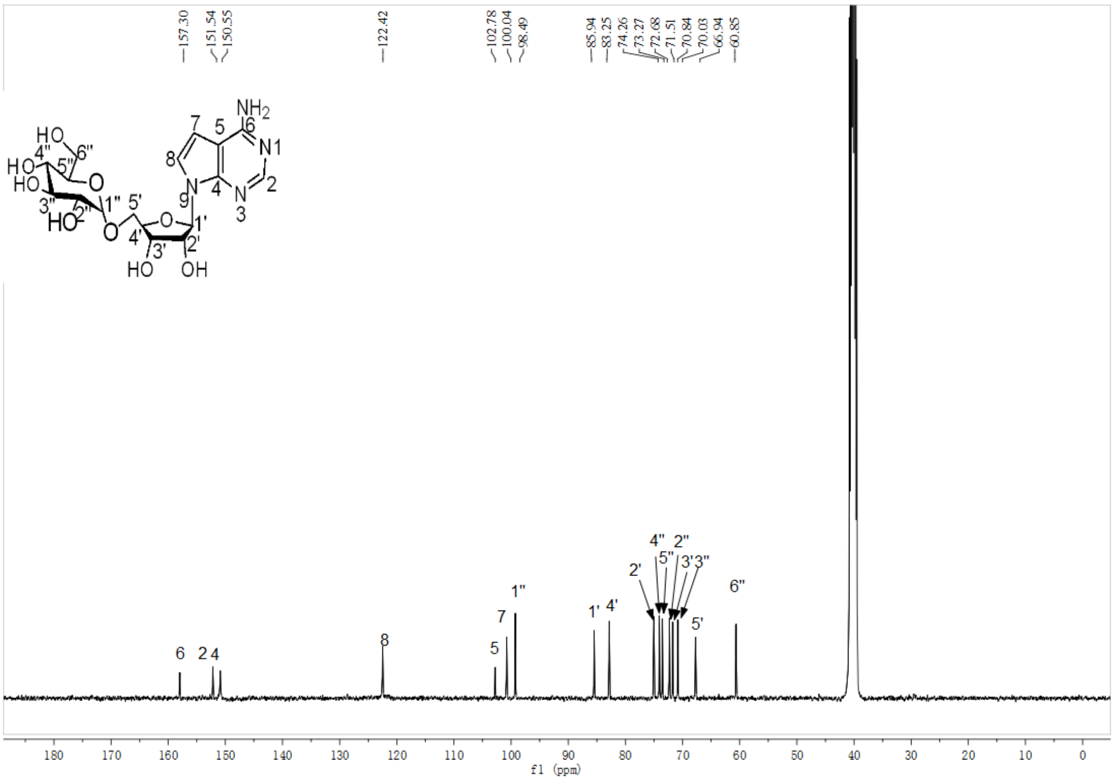

^{13}C NMR (DMSO) of **2**.

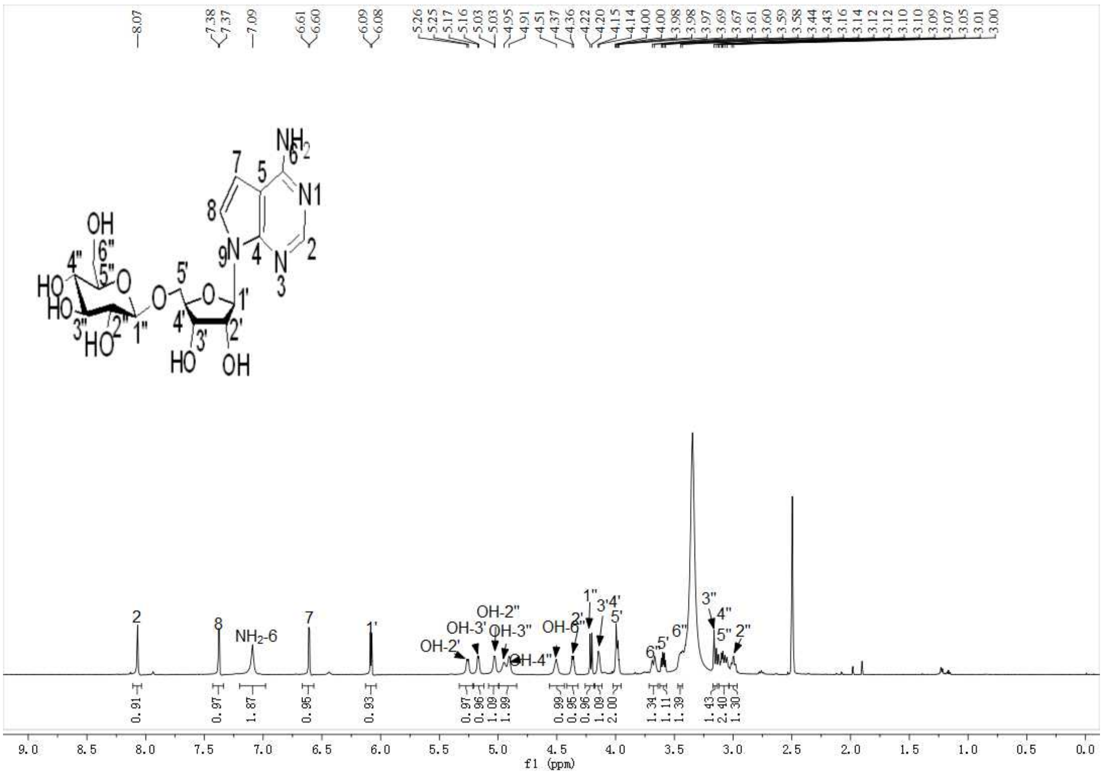

^{1}H NMR (DMSO) of **17**.

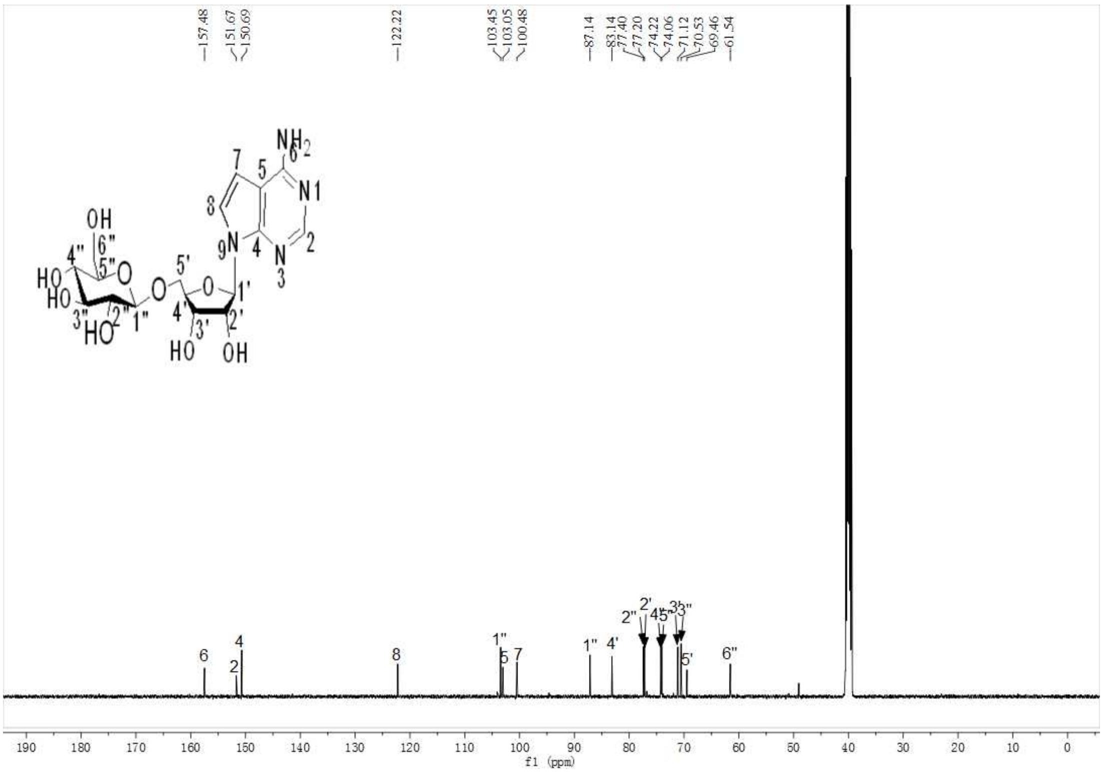

^{13}C NMR (DMSO) of **17**.
